# Synergistic Regulation of S-Vacancy of MoS_2_-Based Materials for Highly Efficient Electrocatalytic Hydrogen Evolution

**DOI:** 10.3389/fchem.2022.915468

**Published:** 2022-06-08

**Authors:** Xiao-Yun Li, Shao-Ju Zhu, Yi-Long Wang, Tian Lian, Xiao-yu Yang, Cui-Fang Ye, Yu Li, Bao-Lian Su, Li-Hua Chen

**Affiliations:** ^1^ State Key Laboratory of Silicate Materials for Architectures, Wuhan University of Technology, Wuhan, China; ^2^ State Key Laboratory of Advanced Technology for Materials Synthesis and Processing, Wuhan University of Technology, Wuhan, China; ^3^ School of Chemistry, Chemical Engineering and Life Science, Wuhan University of Technology, Wuhan, China; ^4^ Department of Histology and Embryology, Tongji Medical College, Huazhong University of Science and Technology, Wuhan, China

**Keywords:** S-vacancy of MoS_2_, heterogeneous interfaces, exposed Mo atoms, electrocatalysis, hydrogen evolution reaction

## Abstract

Low or excessively high concentration of S-vacancy (*C*_S-vacancy_) is disadvantageous for the hydrogen evolution reaction (HER) activity of MoS_2_-based materials. Additionally, alkaline water electrolysis is most likely to be utilized in the industry. Consequently, it is of great importance for fine-tuning *C*_S-vacancy_ to significantly improve alkaline hydrogen evolution. Herein, we have developed a one-step Ru doping coupled to compositing with CoS_2_ strategy to precisely regulate *C*_S-vacancy_ of MoS_2_-based materials for highly efficient HER. In our strategy, Ru doping favors the heterogeneous nucleation and growth of CoS_2_, which leads to a high crystallinity of Ru-doped CoS_2_ (Ru-CoS_2_) and rich heterogeneous interfaces between Ru-CoS_2_ and Ru-doped MoS_2-x_ (Ru-MoS_2-x_). This facilitates the electron transfer from Ru-CoS_2_ to Ru-MoS_2-x_, thereby increasing *C*_S-vacancy_ of MoS_2_-based materials. Additionally, the electron injection effect increases gradually with an increase in the mass of Co precursor (*m*_Co_), which implies more S^2-^ leaching from MoS_2_ at higher *m*_Co_. Subsequently, *C*_S-vacancy_ of the as-synthesized samples is precisely regulated by the synergistic engineering of Ru doping and compositing with CoS_2_. At *C*_S-vacancy_ = 17.1%, a balance between the intrinsic activity and the number of exposed Mo atoms (EMAs) to boost highly active EMAs should be realized. Therefore, the typical samples demonstrate excellent alkaline HER activity, such as a low overpotential of 170 mV at 100 mA cm^−2^ and a TOF of 4.29 s^−1^ at -0.2 V. Our results show promise for important applications in the fields of electrocatalysis or energy conversion.

## Graphical Abstract

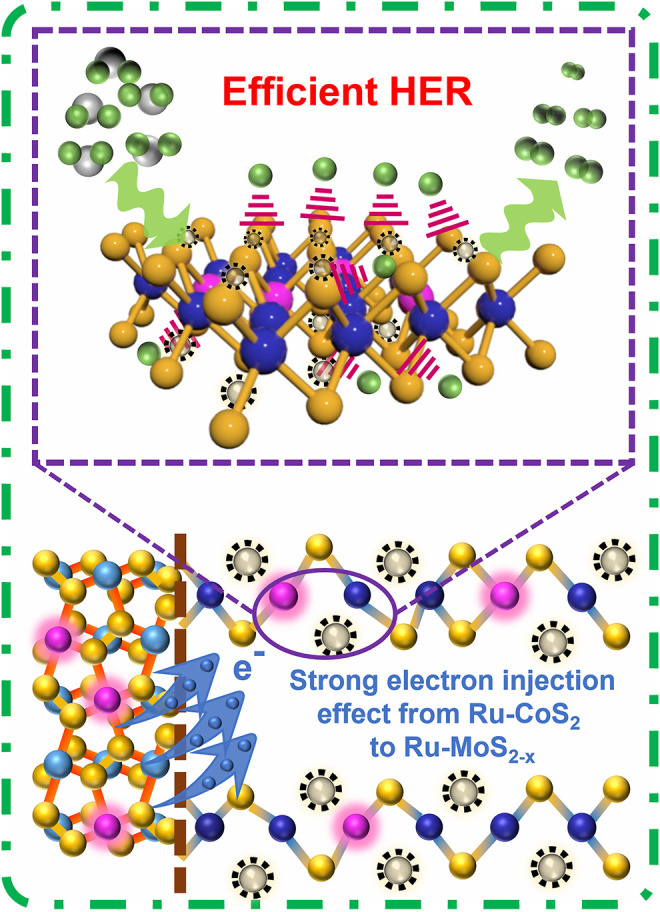

## Introduction

The rapid development of the economy has made the fast consumption of fossil energy, resulting in the energy crisis and severe environmental pollution (Xie et al., 2013b; Xu et al., 2017; Jiang et al., 2022). Hydrogen energy, as one of the clean and renewable energies, has received extensive attention around the world (Huang et al., 2019; Venkateshwaran and Senthil Kumar, 2019; Zhu et al., 2019; Wang F. et al., 2020). Electrocatalytic water splitting (Lin et al., 2017; Lin et al., 2019; Djara et al., 2020; Liu L. et al., 2022) is regarded as an eco-friendly technology for hydrogen (H_2_) production. So far, platinum-based materials are still the best electrocatalysts for acidic water electrolysis (Chen et al., 2020). Nevertheless, the high cost and low abundance seriously restrict the wide applications of such precious metals (Li et al., 2011; Qi et al., 2019; Jing et al., 2020). On the other hand, alkaline water electrolysis is most likely to be utilized in the industry, owing to the unrestricted reactant availability, desirable safety, and satisfactory output (Yin et al., 2015). However, the sluggish dynamics of hydrogen evolution reaction (HER) in alkaline environments results in excessive energy consumption (Yin et al., 2015). Therefore, it is of great significance for the development of highly efficient and low-cost electrocatalysts to overcome energy barriers for accelerating kinetics and to decrease overpotential during the hydrogen evolution reaction (HER) process.

It is well known that molybdenum disulfide (MoS_2_) can be designed as an alternative to Pt due to the excellent H* adsorption–desorption properties, special layered structures, good stability, and low cost (Hinnemann et al., 2005; Deng et al., 2017; Wang Y. et al., 2019; Gao et al., 2020). However, the pristine 2H-MoS_2_ tends to aggregate under van der Waals forces, resulting in poor edge active sites (Kibsgaard et al., 2012; Wang et al., 2017; Wang et al., 2021b). More importantly, there remain a lot of active sites from the vast basal planes of such MoS_2_ to be developed (Kibsgaard et al., 2012; Wang et al., 2017; Wang et al., 2021b). To address these drawbacks, defect-rich, double-gyroid, and amorphous structures have been introduced into such MoS_2_ nanosheets to increase the unsaturated sulfur atoms as active sites for HER (Kibsgaard et al., 2012; Xie et al., 2013a; Liu et al., 2018). Doping transition metals (TMs) into MoS_2_ is another important method for the advancement of electrocatalysis. This is because doping TMs not only increases the number of unsaturated sulfur atoms but also regulates the adsorption free energy of hydrogen atoms (ΔG_H_) of active sites to favor HER (Wang et al., 2015; Wang D. et al., 2019; Wang et al., 2021b). Among these TMs, ruthenium (Ru) belongs to the Pt group, but its price is as low as about 5% of Pt (Zhang J. et al., 2019). A quintessential example demonstrates that ΔG_H_ at the Ru-doped in-plane sulfur sites decreases to approximately 0.19 eV (Zhang X. et al., 2019). This indicates that doping Ru can efficiently modulate the electronic features of the adjacent sulfur atoms, thereby leading to the optimal H atom binding energy (Yan et al., 2018). On the other hand, after doping Ru into 2H-MoS_2_ (Li J. et al., 2021) or 2H-WS_2_ (Li J. et al., 2022), these Ru sites can significantly accelerate the water dissociation in alkaline environments. For example, the energy barrier of the H−OH cleavage (*E*) is as high as 2.42 eV before doping Ru. Remarkably, *E* of such Ru sites decreases to 2.02 eV, which is advantageous for water dissociation to OH and H intermediates.

Modulating S-vacancy into MoS_2_-based materials has been developed as an efficient strategy (Tsai et al., 2017; Park et al., 2018; Li Y. et al., 2021) to activate inert basal planes because the exposed Mo atoms can be tailored into newborn active sites (Wang X. et al., 2020). Following this opinion, much effort has so far been devoted to introducing S-vacancy into the basal planes of mono-layered or multi-layered 2H-MoS_2_ nanosheets (Li et al., 2016b; Tsai et al., 2017; Park et al., 2018; Li et al., 2019; Wang X. et al., 2020). Among these strategies, chemical vapor deposition coupled to plasma has been developed to modulate S-vacancy into MoS_2_ (Li et al., 2016b). In this case, S atoms escape from the MoS_2_ lattice more easily than Mo atoms due to the lower formation energy of S-vacancy compared to that of the Mo interstitial (Li et al., 2016b). Subsequently, the controllable electrochemical preparation of S-vacancy for multi-layered MoS_2_ has been proposed by simply adjusting desulfurization parameters, such as desulfurization potential and time (Tsai et al., 2017). More recently, the single Ru atom doping technology promotes the phase transition of 2H-MoS_2_ and the formation of S-vacancy, which greatly enhances its HER activity (Zhang J. et al., 2019). Nevertheless, a low or excessively high concentration of S-vacancy (*C*_S-vacancy_) is disadvantageous for the hydrogen evolution reaction (HER) activity of MoS_2_-based materials. Consequently, it is of great importance for the development of a novel approach to fine-tuning *C*_S-vacancy_ to significantly improve alkaline hydrogen evolution. In addition, the aforementioned electrocatalysts based on S-vacancy only consist of a single component (that is, MoS_2_) rather than hybrid catalysts. As one of the traditional semiconductor materials, MoS_2_ suffers from another drawback of unsatisfactory charge-transfer resistance (*R*_CT_), leading to low HER activity (Li Y. et al., 2021; Wang et al., 2021b; Wang et al., 2021c). Hence, compositing with the metallic phase will favor fast electrode kinetics, realizing synergistically regulating *C*_S-vacancy_ and *R*_CT_ of MoS_2_-based electrocatalysts for highly efficient HER.

Herein, we develop a one-step Ru doping coupled to compositing with the CoS_2_ strategy to synergistically regulate *C*_S-vacancy_ of MoS_2_-based materials for highly efficient alkaline HER. Ru doping is advantageous for the formation of S-vacancy in the basal planes of MoS_2_. On the other hand, Ru doping favors heterogeneous nucleation and growth of CoS_2_, which leads to rich heterogeneous interfaces between Ru-doped CoS_2_ (Ru-CoS_2_) and Ru-doped MoS_2-x_ (Ru-MoS_2-x_). This facilitates the electron transfer from Ru-CoS_2_ to Ru-MoS_2-x_, thereby increasing *C*_S-vacancy_ of MoS_2_-based materials. At fixed Ru dopant, the electron injection effect increases gradually with an increase in the mass of Co precursor, which means more S^2-^ escaping from Ru-MoS_2_ nanosheets. Therefore, synergistically regulating *C*_S-vacancy_ of the as-synthesized samples, from 2.1 to 27.5%, is realized by a new one-step Ru doping coupled to compositing with the CoS_2_ strategy. On regulating *C*_S-vacancy_ to 17.1%, a balance between the intrinsic activity and the number of exposed Mo atoms (EMAs) to boost highly active EMAs should be realized. As a consequence, the typical samples demonstrate the optimal alkaline HER activity among all samples, such as a low overpotential of 170 mV at 100 mA cm^−2^, a large specific alkaline HER current density of 77.6 μA cm^−2^, and a turnover frequency of 4.29 s^−1^ at -0.2 V as well as excellent long-term stability. Our results pave a novel approach to unlocking the potential of inert basal planes in MoS_2_-based materials for highly efficient HER and promise important applications in the field of electrocatalytic hydrogen evolution.

## Experimental Section

### Fabrication of the Typical Samples (Ru-MoS_2-X_-CoS_2_/CC)

The typical samples were synthesized by a one-pot hydrothermal strategy using the following precursors such as Na_2_MoO_4_.2H_2_O, CH_4_N_2_S, and Co(NO_3_)_2_·6H_2_O. In the typical experiments, 160 mg Co(NO_3_)_2_·6H_2_O, 160 mg Na_2_MoO_4_.2H_2_O, and 600 mg CH_4_N_2_S were dissolved in 46.0 ml deionized water under magnetic stirring. Subsequently, 4.0 ml RuCl_3_ solution (5 mmol L^−1^) was introduced into the above cobalt salt solution under magnetic stirring for 0.5 h. Carbon cloth (CC, 4 cm^2^) was pretreated according to the related literature (Yu et al., 2015). Then, the above solution and the pretreated CC were transferred into a 100.0 ml Teflon-lined stainless-steel autoclave and heated to 200°C for 20 h. After the hydrothermal reaction, the mixture was cooled to room temperature. The typical samples were harvested after being washed with water thoroughly and vacuum-dried at 60 °C for 12.0 h and abbreviated as Ru-MoS_2-x_-CoS_2_/CC.

### Fabrications of Other Ru-MoS_2-X_-CoS_2_/CC Samples

Other Ru-MoS_2-x_-CoS_2_/CC samples were synthesized at various volumes of RuCl_3_ solution (5 mmol L^−1^) of 1.0, 7.0, 10.0, and 30.0 ml under otherwise similar conditions as the typical experiments. These Ru-MoS_2-x_-CoS_2_/CC samples are denoted as Ru-MoS_2-x_-CoS_2_/CC-1.0, Ru-MoS_2-x_-CoS_2_/CC-7.0, Ru-MoS_2-x_-CoS_2_/CC-10.0, and Ru-MoS_2-x_-CoS_2_/CC-30.0.

In addition, Ru-MoS_2-x_-CoS_2_/CC-80, Ru-MoS_2-x_-CoS_2_/CC-240, Ru-MoS_2-x_-CoS_2_/CC-280, and Ru-MoS_2-x_-CoS_2_/CC-320 were synthesized at various masses of Co(NO_3_)_2_·6H_2_O of 80, 240, 280, and 320 mg under otherwise similar conditions of the typical experiments, respectively.

**FIGURE 1:**
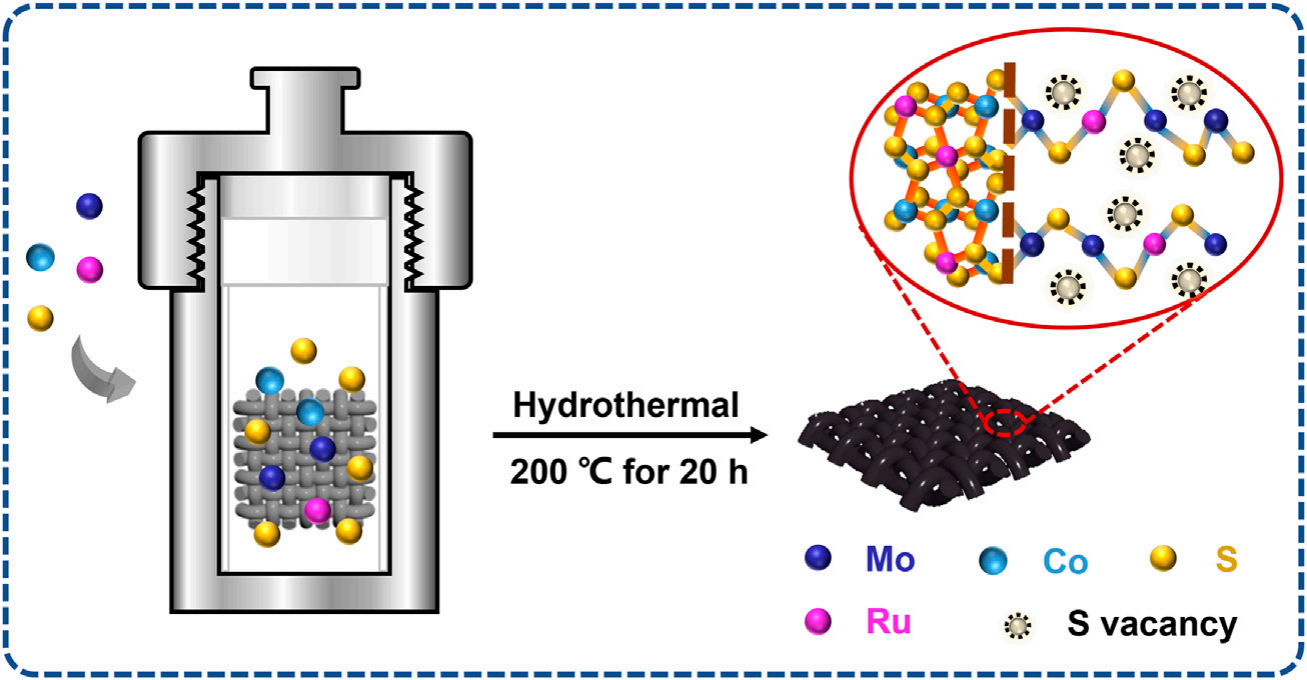
Schematic representation of the fabrication of Ru-MoS_2_-CoS_2_/CC samples.

Figure 1 displays a schematic representation of the fabrication of all Ru-MoS_2-x_-CoS_2_/CC samples.

### Fabrications of Ru-MoS_2_/CC, MoS_2_/CC, MoS_2_-CoS_2_/CC, and Ru-CoS_2_/CC

Ru-doped MoS_2_ nanosheets assembled on CC are abbreviated as Ru-MoS_2_/CC. Fabrication of Ru-MoS_2_/CC is almost the same as the typical samples except for the absence of Co(NO_3_)_2_.

MoS_2_ nanosheets assembled on CC are abbreviated as MoS_2_/CC. Fabrication of MoS_2_/CC is almost the same as Ru-MoS_2_/CC except for the absence of RuCl_3_ solution.

MoS_2_ nanosheets coated with CoS_2_ assembled on CC are abbreviated as MoS_2_-CoS_2_/CC. Fabrication of MoS_2_-CoS_2_/CC is almost the same as the typical samples except for the absence of RuCl_3_ solution.

Ru-doped CoS_2_ assembled on CC are abbreviated as Ru-CoS_2_/CC. Fabrication of Ru-CoS_2_/CC is almost similar to the typical samples except for the absence of Na_2_MoO_4_.2H_2_O.

Other experimental details about materials, characterization, and performance measurements are supplied in Supplementary Material S1.

## Results and Discussion

### Characterization of the Typical Samples

**FIGURE 2:**
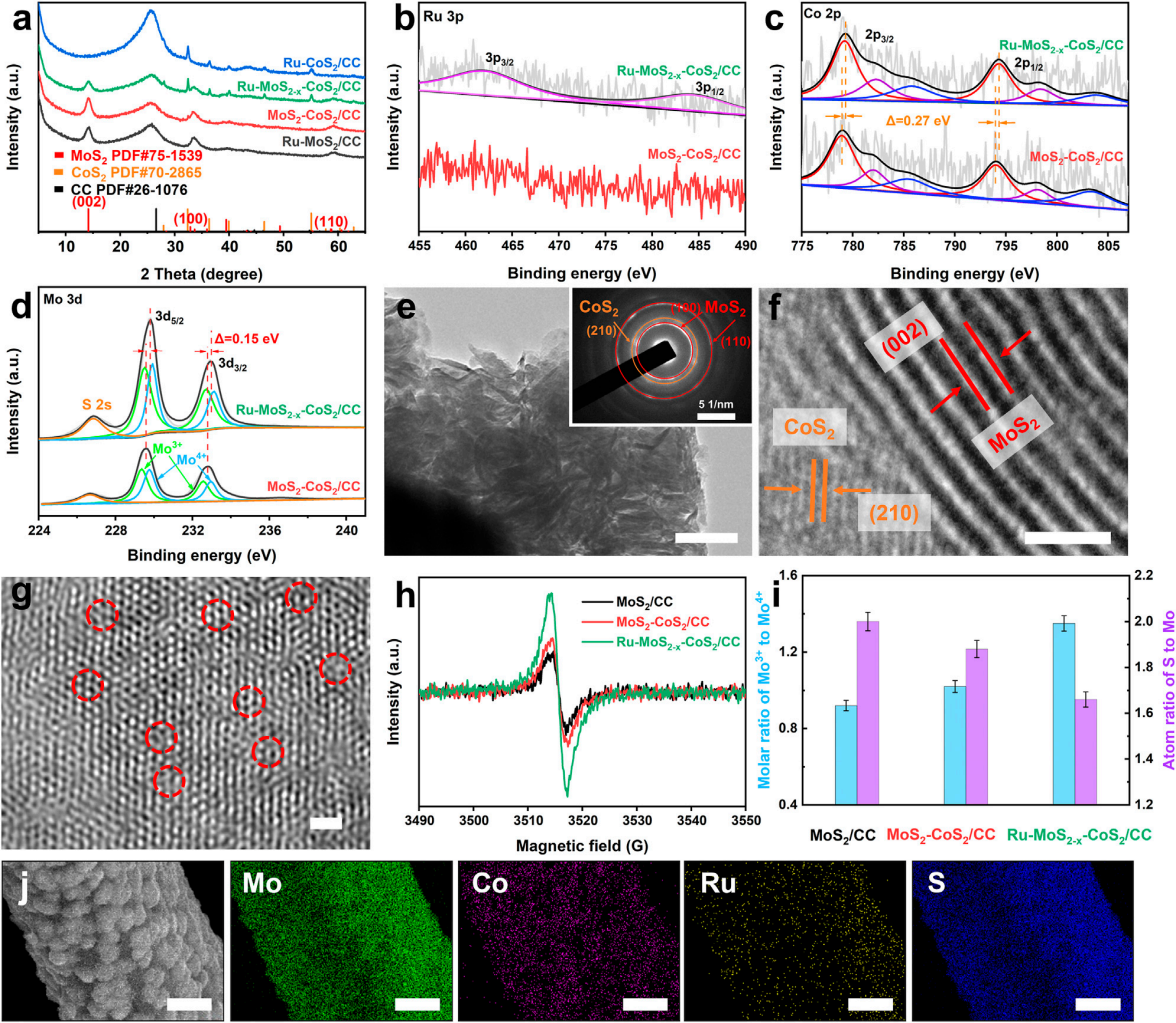
**(A)** XRD patterns of Ru-MoS_2_/CC, MoS_2_-CoS_2_/CC, Ru-CoS_2_/CC, and the typical samples; **(B)** Ru 3p, **(C)** Co 2p, and **(D)** Mo3d high-resolution XPS spectra of the typical samples and MoS_2_-CoS_2_/CC; **(E)** TEM image and **(F,G)** HR-TEM images of the typical samples; **(H)** EPR spectra, and **(I)** molar ratio of Mo^3+^ to Mo^4+^, and the atom ratio of S to Mo of MoS_2_/CC, MoS_2_-CoS_2_/CC, and the typical samples. **(J)** EDX mapping profiles of Mo, Co, Ru, and S over the typical samples. The inset of panel **(E)** is the SAED pattern of the typical samples. Scale bars of panels **(E)**, **(F)**, **(G)**, and **(J)** are 100 nm, 2 nm, 2 nm, and 2 μm, respectively.

Scanning electron microscopic (SEM) images display the morphology of the typical samples in Supplementary Figures SJ–L. From these figures, it can be observed that lots of nanosheets are grown on the smooth surface of CC (Supplementary Figure S2). Powder X-ray diffraction (XRD) pattern of Ru-MoS_2-x_-CoS_2_/CC (Figure 2A) shows the diffraction peaks located at 2*θ* = 14.1°, 32.7°, and 58.8°, matching well with the (002), (100), and (110) planes of 2H-MoS_2_ (PDF#75-1539) (Nguyen et al., 2021), respectively. Other sharp diffraction peaks of Ru-MoS_2-x_-CoS_2_/CC, such as 32.4° and 55.1°, are attributed to the (200) and 311) planes of CoS_2_ (PDF#70-2865) (Yao et al., 2019), respectively, suggesting the presence of highly crystallized CoS_2_ besides 2H-MoS_2_. From Figure 2A, we also observe the diffraction peak at 26.2° originating from CC. The Raman spectrum of Ru-MoS_2-x_-CoS_2_/CC (Supplementary Figure S3) displays the typical *E* ^1^_2g_ and *A*_1g_ vibration models of the Mo-S bonds, further verifying the phase structure of 2H-MoS_2_ in Ru-MoS_2-x_-CoS_2_/CC. As shown in Figure 2B, the Ru 3p high-resolution X-ray photoelectron spectroscopy (XPS) spectrum of Ru-MoS_2-x_-CoS_2_/CC is divided into two characteristic peaks at the binding energies of 462.4 and 484.7 eV, corresponding to Ru 3p_3/2_ and Ru 3p_1/2_ (Ge et al., 2022). Nevertheless, no peak of Ru is detected in MoS_2_ nanosheets coated with CoS_2_ assembled on CC (MoS_2_-CoS_2_/CC) in Figure 2B. Furthermore, Figures 2C, D exhibit Co 2p and Mo 3 day XPS spectra of Ru-MoS_2-x_-CoS_2_/CC, respectively. It is seen that Co 2p and Mo 3 day peaks positively shift by about 0.27 and 0.15 eV compared to those of MoS_2_-CoS_2_/CC (Hao et al., 2017), respectively. Additionally, the characteristic peaks about Ru_2_S_3_ or RuCl_3_ are not observed in the XRD pattern of Ru-MoS_2-x_-CoS_2_/CC (Figure 2A). Therefore, these data confirm the successful doping of Ru into the following two phases of Ru-MoS_2-x_-CoS_2_/CC, 2H-MoS_2_, and CoS_2_.

Nanosheets of Ru-MoS_2-x_-CoS_2_/CC are also clearly observed from transmission electron microscopic (TEM) image (Figure 2E), which is consistent with the results of SEM images. According to the high-resolution TEM (HR-TEM) image (Figure 2F), the lattice spacings of 0.625 and 0.245 nm that are seen in Ru-MoS_2-x_-CoS_2_/CC correspond to the (002) plane of MoS_2_ and the (210) plane of CoS_2_ (He et al., 2020; Liu X. et al., 2022), respectively. These also conform to the results of XRD. As we know, MoS_2_ is regarded as one of the traditional semiconductor materials, and CoS_2_ is a metallic phase due to its high Fermi level. Therefore, there are lots of Schottky heterojunctions among Ru-MoS_2-x_-CoS_2_/CC. In addition, the inset of Figure 2E (selected area electron diffraction, SAED) pattern indicates that Ru-doped MoS_2_ (Ru-MoS_2_) and Ru-doped CoS_2_ (Ru-CoS_2_) of Ru-MoS_2-x_-CoS_2_/CC are polycrystalline (Huang et al., 2015). We can clearly observe that many defects exist in such Ru-MoS_2_ from Figure 2G due to lattice distortion by Ru doping (Zhang J. et al., 2019) and the electron injection effect (Gan et al., 2018; Zhang J. et al., 2019). Energy-dispersive X-ray (EDX) spectroscopy mapping (Figure 2J) profiles further confirm the uniform distribution of Mo, Co, Ru, and S elements throughout Ru-MoS_2-x_-CoS_2_/CC. Considering that Ru-MoS_2_ assembled on CC (Ru-MoS_2_/CC) and Ru-CoS_2_ assembled on CC (Ru-CoS_2_/CC) tend to form nanosheets (Supplementary Figures S1B, C) and nanoparticles (Supplementary Figures SH, I), respectively, and that Ru-CoS_2_ nucleates and grows prior to Ru-MoS_2_ (Figures 4A–F), we can reasonably deduce as follows: during the hydrothermal process, Ru-CoS_2_ nanoparticles are firstly assembled on CC; then, Ru-CoS_2_/CC are densely coated by Ru-MoS_2_ nanosheets to construct the typical samples, Ru-MoS_2-x_-CoS_2_/CC.

According to XPS data in Figure 2D and Supplementary Figure S4, we characterize molar ratios of Mo^3+^ to Mo^4+^ of MoS_2_/CC, MoS_2_-CoS_2_/CC, and Ru-MoS_2-x_-CoS_2_/CC in Figure 2I. The presence of Mo^3+^ can induce S-vacancy of the basal planes in Ru-MoS_2_ (Ma et al., 2020) of Ru-MoS_2-x_-CoS_2_/CC. From Figure 2I, Ru-MoS_2-x_-CoS_2_/CC exhibits a higher value of about the molar ratio of Mo^3+^ to Mo^4+^ than MoS_2_/CC or MoS_2_-CoS_2_/CC, reaching 1.35. To confirm such defective structures, electron paramagnetic resonance (EPR) (Liu et al., 2017; Gong et al., 2020; Wang J. et al., 2021) is further employed to estimate the S-vacancy of all samples. As expected, Ru-MoS_2-x_-CoS_2_/CC demonstrates the highest EPR signal at g = ∼ 2.002 among all samples (Figure 2H). Moreover, the EPR signal of MoS_2_-CoS_2_/CC is higher than that of MoS_2_/CC. These data straightforwardly indicate that the formation of S-vacancy might be closely related to both Ru doping into MoS_2_ and compositing with CoS_2_.

### Doping Ru Coupled to Compositing With CoS_2_ to Regulate Microstructures of the As-synthesized Samples

**FIGURE 3:**
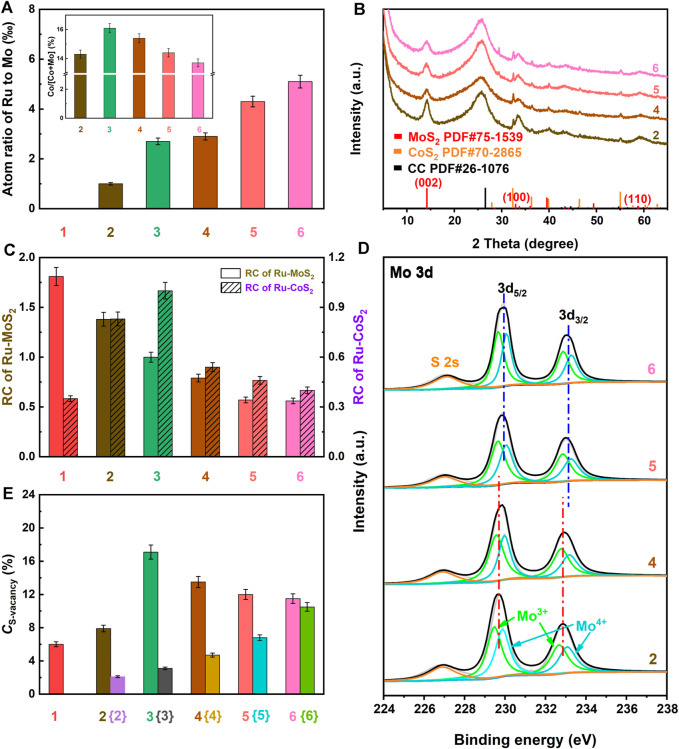
**(A)** Atom ratio of Ru to Mo based on ICP-OES, **(B)** XRD patterns, **(C)** RC of Ru-MoS_2-x_ and Ru-CoS_2_, **(D)** Mo3d high-resolution XPS spectra, and **(E)** *C*_S-vacancy_ of the related samples. Meanwhile, 1, 2, 3, 4, 5 and 6 are denoted as MoS_2_-CoS_2_/CC, Ru-MoS_2-x_-CoS_2_/CC-1.0, the typical samples, Ru-MoS_2-x_-CoS_2_/CC-7.0, Ru-MoS_2-x_-CoS_2_/CC-10.0, and Ru-MoS_2-x_-CoS_2_/CC-30.0, respectively. Fabrications of {2}, {3}, {4}, {5}, and {6} are the same as those of Ru-MoS_2-x_-CoS_2_/CC-1.0, the typical samples, Ru-MoS_2-x_-CoS_2_/CC-7.0, Ru-MoS_2-x_-CoS_2_/CC-10.0, and Ru-MoS_2-x_-CoS_2_/CC-30.0 except for the absence of Co(NO_3_)_2_, to be denoted as Ru-MoS_2_/CC-1.0, Ru-MoS_2_/CC, Ru-MoS_2_/CC-7.0, Ru-MoS_2_/CC-10.0, and Ru-MoS_2_/CC-30.0, respectively. The inset of panel **(A)** is the atom ratio of Co to (Co + Mo) of the related samples based on ICP-OES.

First, a series of Ru-MoS_2_/CC samples were synthesized at the various volumes of RuCl_3_ solution (*V*) of 1.0, 4.0, 7.0, 10.0, and 30.0 ml under otherwise similar conditions of the typical experiments except for the absence of Co(NO_3_)_2_. These Ru-MoS_2_/CC samples are denoted as Ru-MoS_2_/CC-1.0, Ru-MoS_2_/CC-7.0, Ru-MoS_2_/CC-10.0, and Ru-MoS_2_/CC-30.0. Similar Ru 3p XPS spectra to the typical samples are observed in Supplementary Figure S5A, which is responsible for successfully doping Ru into all Ru-MoS_2_/CC samples. In addition, the atom ratios of Ru to Mo (*A*) of all Ru-MoS_2_/CC samples are characterized by XPS in Supplementary Figure S5B. From this figure, *A* increases with increasing *V*, indicating that more Ru will be doped into the Ru-MoS_2_/CC samples at higher *V*. In this work, the atomic ratio of S to Mo (S: Mo) of MoS_2_/CC is firstly measured (Supplementary Table S1) and is normalized to 2.00. Then, it is employed as a reference to confirm the normalized S: Mo of MoS_2-x_ in all Ru-MoS_2_/CC samples in terms of XPS data (Xu et al., 2016; Wang J. et al., 2021). Here, the measured S: Mo for all Ru-MoS_2_/CC samples are the atom ratios of S minus double Ru to Mo, which is abbreviated as [(S–2Ru)/Mo]. Therefore, the normalized S: Mo of MoS_2-x_ in all samples are listed in Supplementary Table S2. From Figure 3E, *C*_S-vacancy_ of Ru-MoS_2_/CC samples increases with the increase in Ru dopants. For example, *C*_S-vacancy_ of Ru-MoS_2_/CC-30.0 increases to 10.5% at *V* = 30.0 ml. That is to say, doping Ru can regulate *C*_S-vacancy_ of Ru-MoS_2_/CC samples varying from 2.1 to 10.5%.

Density functional theory (DFT) calculation reveals that the Ru-S bonding energy (about 0.92 eV) decreases by 0.87 eV compared to the Mo-S bonding energy (about 1.79 eV) (Luo et al., 2020), which is advantageous for the formation of S-vacancy in the basal planes of MoS_2_. In this work, Ru atoms substituting for Mo atoms in MoS_2_ are abbreviated as Ru_(Mo)_. According to the previously reported literature (Zhang J. et al., 2019; Zhang X. et al., 2019), Ru doping results in an increase in *C*_S-vacancy_ due to the strong attractive interaction between Ru_(Mo)_ and the adjacent S-vacancy. Furthermore, S-vacancy is exclusively stable around Ru_(Mo)_ (Kang, 2021).

To investigate the synergistic effect of doping Ru coupled to compositing with CoS_2_ on the microstructure, other Ru-MoS_2-x_-CoS_2_/CC samples are further synthesized at various *V* of 1.0, 7.0, 10.0, and 30.0 ml under otherwise similar conditions of the typical experiments to be denoted as Ru-MoS_2-x_-CoS_2_/CC-1.0, Ru-MoS_2-x_-CoS_2_/CC-7.0, Ru-MoS_2-x_-CoS_2_/CC-10.0, and Ru-MoS_2-x_-CoS_2_/CC-30.0, respectively. According to Supplementary Figure S6, these Ru-MoS_2-x_-CoS_2_/CC samples almost display the same Ru 3p XPS spectra as the typical samples, indicating a successful doping of Ru into them as well. The values of *A* of all samples are characterized by inductively coupled plasma-optical emission spectroscopy (ICP-OES). In terms of Figure 3A, the values of *A* of these samples increase with the increase in *V*. Moreover, other Ru-MoS_2-x_-CoS_2_/CC samples also exhibit the same phase structure as the typical samples, since the XRD pattern of each sample is similar to that of the typical samples (Figure 3B). Figure 3C further presents the relative crystallinity (RC) of Ru-MoS_2-x_ and Ru-CoS_2_ for all Ru-MoS_2-x_-CoS_2_/CC samples based on the corresponding XRD data such as the (002) plane for molybdenum disulfide and (200) (210), (211) (220), and 311) planes for cobalt disulfide. From this figure, the RC of Ru-MoS_2-x_ decreases with the increase in *V*, which implies that more defective structures or S-vacancy would be modulated into the basal planes of Ru-MoS_2-x_ at higher *V*. However, the influence of *V* on RC of Ru-CoS_2_ is not coincident with the trend of the former. Initially, the RC of Ru-CoS_2_ gradually increases with increasing *V*. At *V* = 4.0 ml, the RC of Ru-CoS_2_ of the typical samples exhibits the highest value among all samples. However, further increasing *V* leads to a decrease in the RC of Ru-CoS_2_.

In addition, Mo^3+^ can be observed in Mo 3 day XPS spectra of all Ru-MoS_2-x_-CoS_2_/CC samples (Figure 3D), implying the formation of S-vacancy of the basal planes in Ru-MoS_2-x_. Taking into account that MoS_2_-CoS_2_/CC and other Ru-MoS_2-x_-CoS_2_/CC samples (Supplementary Figures S1E, F; Supplementary Figure S7) are almost similar to the morphology of the typical samples (Supplementary Figures S1K, L), such core–shell structure, Ru-MoS_2-x_ nanosheets densely coated Ru-CoS_2_ composites, facilitates to correctly reflect the S: Mo of Ru-MoS_2-x_ in all samples by XPS analysis. As mentioned above, the S: Mo of MoS_2_/CC is also employed as a reference to confirm the normalized S: Mo of Ru-MoS_2-x_ in all Ru-MoS_2-x_-CoS_2_/CC samples (Li et al., 2016a; Xu et al., 2016) based on XPS data (Supplementary Figure S8). Here, the measured S: Mo for all Ru-MoS_2-x_-CoS_2_/CC samples is the atom ratio of S minus double (Co + Ru) to Mo, which is abbreviated as [(S–2Co–2Ru)/Mo]. Subsequently, we further calculate the related *C*_S-vacancy_, as shown in Figure 3E; Supplementary Table S1. From Figure 3E, *C*_S-vacancy_ of Ru-MoS_2-x_-CoS_2_/CC increases to 7.9% compared to that of MoS_2_-CoS_2_/CC (about 6.0%). Out of expectation, *C*_S-vacancy_ of the typical samples rather than Ru-MoS_2-x_-CoS_2_/CC-30.0 reaches the highest value among all samples, about 17.1% from Figure 3E. At the same time, Ru-MoS_2-x_-CoS_2_/CC-30.0 exhibits a lower EPR signal than the typical samples (Supplementary Figure S9), further confirming fewer S-vacancy for Ru-MoS_2-x_-CoS_2_/CC-30.0 in comparison with the typical samples. In other words, at a fixed mass of Co precursor, *C*_S-vacancy_ of Ru-MoS_2-x_ can be precisely regulated from 7.9 to 17.1% by synergistic Ru doping and compositing with CoS_2_ engineering. As a consequence, we can infer that compositing with CoS_2_ should be another key factor to regulate *C*_S-vacancy_ of Ru-MoS_2-x_ nanosheets in Ru-MoS_2-x_-CoS_2_/CC samples. This is because Ru doping also affects the microstructure of CoS_2_, which may have a significant influence on *C*_S-vacancy_ of Ru-MoS_2-x_ nanosheets in Ru-MoS_2-x_-CoS_2_/CC samples in turn.

**FIGURE 4:**
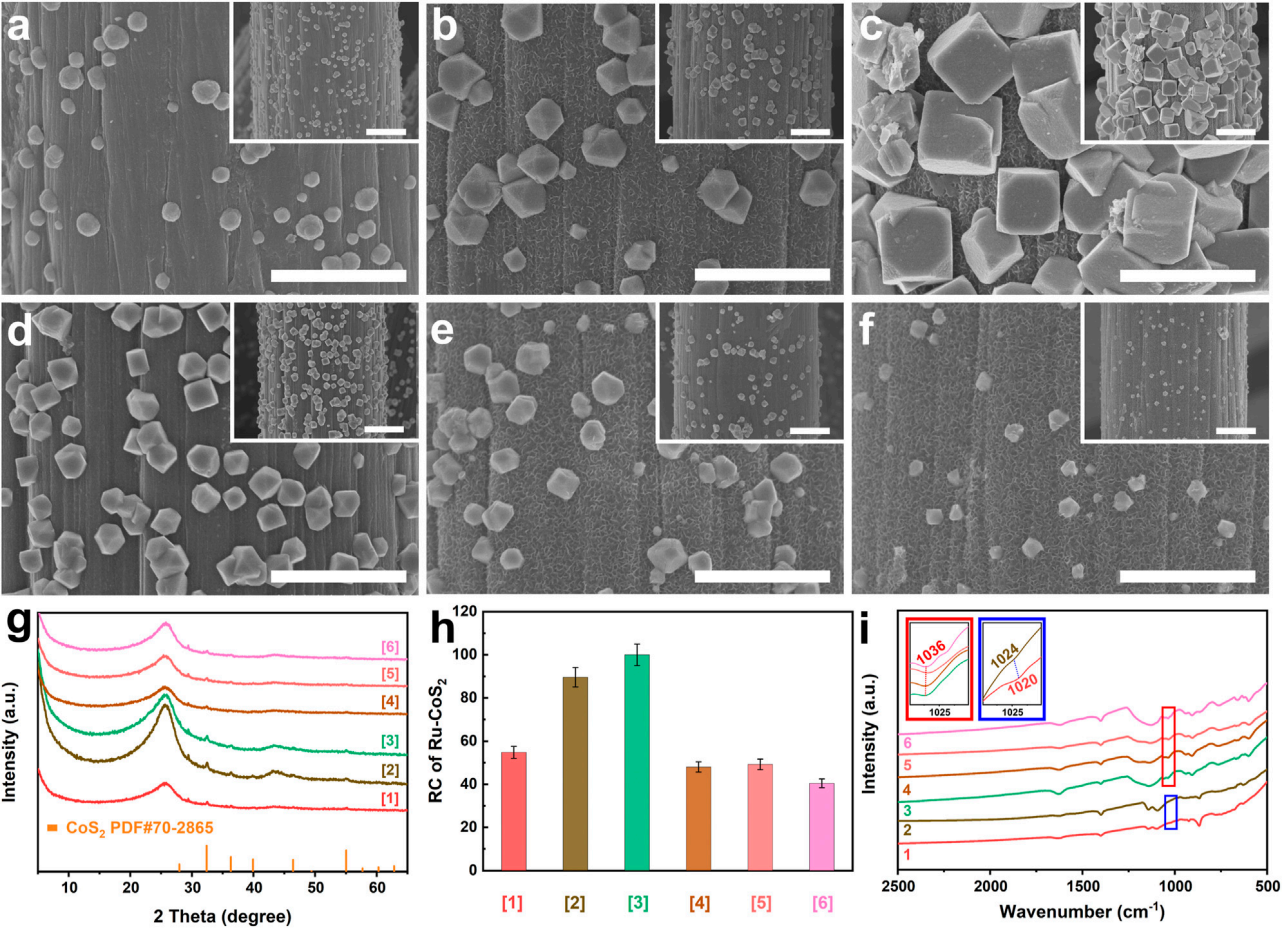
SEM images of **(A)** [MoS_2_-CoS_2_/CC] ([1]), **(B)** [Ru-MoS_2-x_-CoS_2_/CC-1.0] ([2]), **(C)** [the typical samples] ([3]), **(D)** [Ru-MoS_2-x_-CoS_2_/CC-7.0] ([4]), **(E)** [Ru-MoS_2-x_-CoS_2_/CC-10.0] ([5]), and **(F)** [Ru-MoS_2-x_-CoS_2_/CC-30.0] ([6]); **(G)** XRD patterns and **(H)** RC of Ru-CoS_2_ of the corresponding samples; and **(I)** FT-IR spectra of the related samples. Meanwhile, 1, 2, 3, 4, 5, and 6 are MoS_2_-CoS_2_/CC, Ru-MoS_2-x_-CoS_2_/CC-1.0, the typical samples, Ru-MoS_2-x_-CoS_2_/CC-7.0, Ru-MoS_2-x_-CoS_2_/CC-10.0, and Ru-MoS_2-x_-CoS_2_/CC-30.0, respectively. Additionally, [1], [2], [3], [4], [5], and [6] are the corresponding samples synthesized at t = 6 h rather than 20 h. The insets of panels **(A–F)** are the low-magnified SEM images of the corresponding samples. The insets in red and blue solid lines of panel **(I)** are the high-magnified images of panel **(I)** indicated in red and blue solid frames, respectively. Scale bars of panels **(A–F)** and their insets are 2 and 5 μm.

Following this viewpoint, we deeply investigate the influence of doping Ru on the microstructure of cobalt disulfide. As mentioned above, CoS_2_ nucleates and grows prior to MoS_2_. To eliminate the effect of MoS_2_ as much as possible during the characterization of microstructures of CoS_2_, the related samples such as [MoS_2_-CoS_2_/CC], [1] [Ru-MoS_2-x_-CoS_2_/CC], [2], [3], and [4] are synthesized at *t* = 6 h (Figures 4A–F) rather than 20 h. Fabrications of [MoS_2_-CoS_2_/CC], [1] [Ru-MoS_2-x_-CoS_2_/CC], [2], [3], and [4] are the same as those of MoS_2_-CoS_2_/CC, Ru-MoS_2-x_-CoS_2_/CC-1.0, the typical samples, Ru-MoS_2-x_-CoS_2_/CC-7.0, Ru-MoS_2-x_-CoS_2_/CC-10.0, and Ru-MoS_2-x_-CoS_2_/CC-30.0 except for *t*, respectively. Their SEM images and XRD patterns are displayed in Figures 4A–G, respectively. Besides the diffraction peak at 26.2° of CC, other peaks are indexed to CoS_2_ without the signal of MoS_2_ for all samples (Figure 4G), further confirming the heterogeneous nucleation and growth of CoS_2_ prior to MoS_2_. As shown in Figure 4A, a small number of spherical CoS_2_ nanoparticles are grown on CC for [MoS_2_-CoS_2_/CC]. Moreover, the similar phenomena are observed for Ru-doped samples from Figures 4B–F. Interestingly, with an increase in *V*, the size and number of Ru-CoS_2_ nanoparticles gradually increase (Figure 4B). At *V* = 4.0 ml, both the size and number of Ru-CoS_2_ nanoparticles for [Ru-MoS_2-x_-CoS_2_/CC] reach up to the maximum value (Figure 4C). However, further increasing *V* leads to a decrease in both the size and number of Ru-CoS_2_ for other [Ru-MoS_2-x_-CoS_2_/CC] (Figures 4D–F). Next, we characterize the RC of Ru-CoS_2_ for all samples synthesized at 6 h according to XRD data such as (200) (210), (211) (220), and (311) planes of cobalt disulfide, as shown in Figure 4H. Impressively, their trend in the variation is almost consistent with that of the RC of Ru-CoS_2_ of Ru-MoS_2-x_-CoS_2_/CC samples synthesized at 20 h (Figure 3C). This confirms that doping Ru can improve the crystallinity of CoS_2_ under certain conditions. To find out the reason for this issue, we investigate the Fourier-transforming infrared (FT-IR) spectrum of MoS_2_-CoS_2_/CC in Figure 4I. The peak located at 1020 cm^−1^ straightforwardly indicates the formation of CoS_2_ (Chakraborty et al., 2006) at *V* = 0.0 ml. At *V* increasing to 1.0 ml, the peak of Ru-MoS_2-x_-CoS_2_/CC-1.0 located at 1024 cm^−1^ shows a blue-shift of about 4 cm^−1^ compared to that of MoS_2_-CoS_2_/CC. At *V* = 4.0 ml, the related peak blue-shifts to about 1036 cm^−1^, as shown in Figure 4I. This indicates that doping Ru into CoS_2_ can decrease the Co−S bond length and enhance the bonding energy (Feng et al., 2018), which favors the formation of Ru-CoS_2_. However, a decrease in both the size and number of CoS_2_ with further increasing *V* (Figures 4D–F) can be explained as follows: introducing excessive Ru into the lattice of CoS_2_ may induce an increase in the oxidation state of neighboring Co ions to maintain the charge neutrality. The similar result has been observed for other transition metal compounds, such as NiO (Smyth, 2000; Ge et al., 2022). This implies that excessive dopant will hinder the formation of CoS_2_ in turn.

### Possible Formation Mechanism of S-Vacancy in Ru-MoS_2-X_-CoS_2_/CC Samples

**FIGURE 5:**
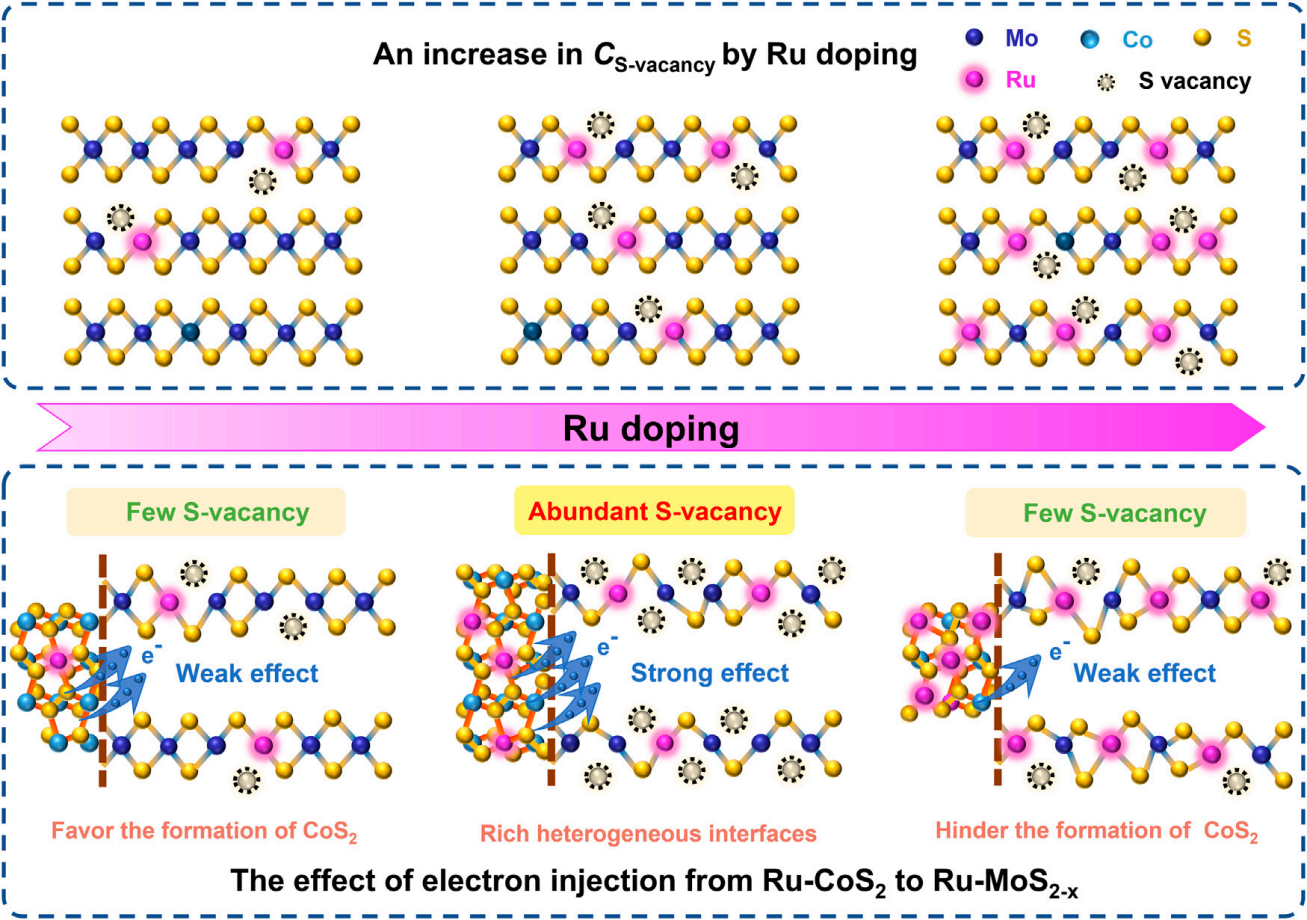
Schematic illustration of the possible formation mechanism of the S-vacancy of the basal planes in Ru-MoS_2-x_ of Ru-MoS_2-x_-CoS_2_/CC samples.

Based on these results and discussion, a possible formation mechanism of S-vacancy of the basal planes in Ru-MoS_2-x_ of Ru-MoS_2-x_-CoS_2_/CC samples can be proposed as follows: on the one side, S-vacancy is modulated into the basal planes of MoS_2_ due to the strong attractive interaction between Ru_(Mo)_ and the adjacent S-vacancy. Furthermore, S-vacancy is exclusively stable around Ru_(Mo)_. On the other hand, by compositing with CoS_2_, Schottky heterojunctions provide a feasible opportunity for the electron transfer from Ru-CoS_2_ to Ru-MoS_2-x_ (Li et al., 2018) due to a high Fermi level of CoS_2_. This effect can promote S^2-^ escaping from Ru-MoS_2-x_ nanosheets to remain charge neutrality (Liu et al., 2017; Gan et al., 2018), indicating the formation of the positively charged defects, S-vacancy. Subsequently, we further characterize the atom ratios of Co to (Co + Mo) of the related samples by ICP-OES in the inset of Figure 3A. From this inset, the atom ratio of Co to (Co + Mo) of the typical samples is as high as about 16.1% at *V* increasing to 4.0 ml, thereby resulting in more Ru-CoS_2_, and rich heterogeneous interfaces between Ru-CoS_2_ and Ru-MoS_2-x_ as well. More heterogeneous interfaces favor the electron injection from Ru-CoS_2_ to Ru-MoS_2-x_, which leads to an increase in *C*_S-vacancy_ (Figure 3E). However, further increasing *V* results in a decrease in atom ratios of Co to (Co + Mo) (inset of Figure 3A) and poor heterojunctions. This could indicate a weaker electron injection effect of Ru-CoS_2_ and lower *C*_S-vacancy_ at excessively high *V*. The schematic illustration of the possible formation mechanism of S-vacancy of the basal planes in Ru-MoS_2-x_ of Ru-MoS_2-x_-CoS_2_/CC samples is presented in Figure 5.

**FIGURE 6:**
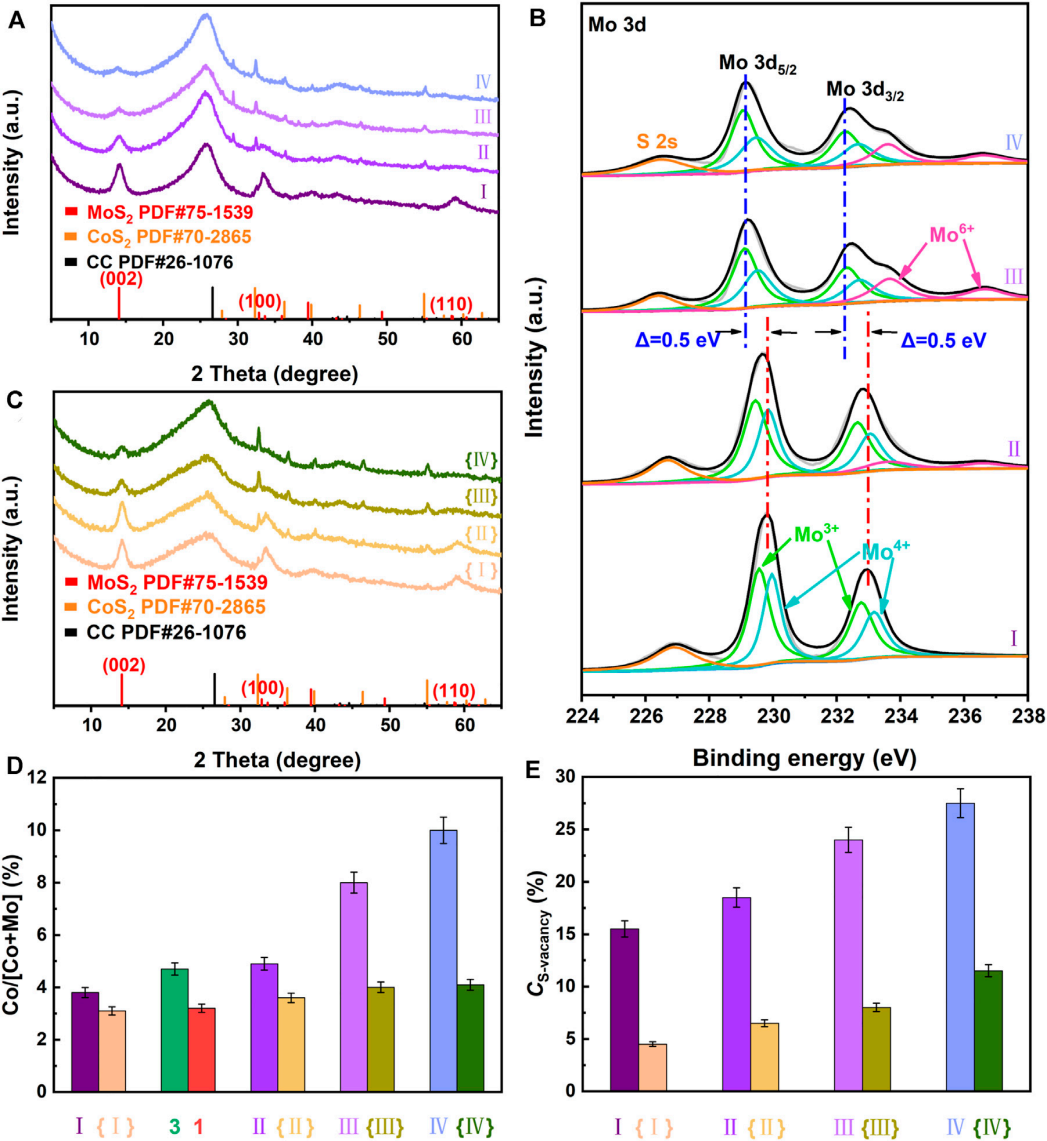
**(A)** XRD patterns and **(B)** Mo3d high-resolution XPS spectra of Ru-MoS_2-x_-CoS_2_/CC-80 (Ⅰ), Ru-MoS_2-x_-CoS_2_/CC-240 (Ⅱ), Ru-MoS_2-x_-CoS_2_/CC-280 (Ⅲ), and Ru-MoS_2-x_-CoS_2_/CC-320 (Ⅳ); **(C)** XRD patterns of MoS_2_-CoS_2_/CC-80 ({Ⅰ}), MoS_2_-CoS_2_/CC-240 ({Ⅱ}), MoS_2_-CoS_2_/CC-280 ({Ⅲ}), and MoS_2_-CoS_2_/CC-320 ({Ⅳ}); **(D)** atom ratios of Co to (Co + Mo) by XPS data, **(E)** *C*_S-vacancy_ of the related samples. Meanwhile, fabrications of MoS_2_-CoS_2_/CC-80, MoS_2_-CoS_2_/CC-240, MoS_2_-CoS_2_/CC-280, and MoS_2_-CoS_2_/CC-320 are almost the same as those of Ru-MoS_2-x_-CoS_2_/CC-80, Ru-MoS_2-x_-CoS_2_/CC-240, Ru-MoS_2-x_-CoS_2_/CC-280, and Ru-MoS_2-x_-CoS_2_/CC-320 except for the absence of RuCl_3_ solution.

To confirm this viewpoint, we systematically investigate the effect of compositing with CoS_2_ on *C*_S-vacancy_ of the as-synthesized samples at fixed *V* or Ru dopants in Figure 6. Figure 6A exhibits XRD patterns of Ru-MoS_2-x_-CoS_2_/CC-80, Ru-MoS_2-x_-CoS_2_/CC -240, Ru-MoS_2-x_-CoS_2_/CC-280, and Ru-MoS_2-x_-CoS_2_/CC-320. With an increase in the mass of Co precursor (*m*_Co_), the intensity of the diffraction peak of the (002) plane at 14.1° for Ru-MoS_2-x_ greatly decreases, while intensities of peaks of the (200) and 311) planes for Ru-CoS_2_ gradually increase (Figure 6A), indicating a decrease in the RC of Ru-MoS_2-x_ and an increase in the RC of Ru-CoS_2_ (Supplementary Figure S10). At *m*_Co_ increasing to 240 mg, Ru-CoS_2_ can be densely coated by Ru-MoS_2-x_ nanosheets (Supplementary Figure S11B). Nevertheless, further increasing *m*_Co_ to 280 or 320 mg leads to incompact Ru-MoS_2-x_ shells (Supplementary Figures S11C, D) due to the formation of more Ru-CoS_2_ or a decrease in the content of Ru-MoS_2-x_. These data are in good agreement with the results of Figure 6D, which displays atom ratios of Co to (Co + Mo) of the as-synthesized samples by XPS analysis. These atom ratios also increase with increasing *m*_Co_. For example, the atom ratio of Co to (Co + Mo) of R Ru-MoS_2-x_-CoS_2_/CC-320 increases up to 10.1%. To further confirm the effect of electron injection from Ru-CoS_2_ to Ru-MoS_2-x_, we characterize Mo3d high-resolution XPS spectra of Ru-MoS_2-x_-CoS_2_/CC-80, Ru-MoS_2-x_-CoS_2_/CC-240, Ru-MoS_2-x_-CoS_2_/CC-280, and Ru-MoS_2-x_-CoS_2_/CC-320 in Figure 6B. Mo 3 days in XPS data move to low binding energy (Huang et al., 2019), implying an increase in the electron density of Ru-MoS_2-x_ of the as-synthesized samples. For example, Mo 3 day peaks of the typical samples negatively shift by about 0.06 eV compared to those of Ru-MoS_2-x_-CoS_2_/CC-80 in terms of Figure 2D and Figure 6B. After careful investigations, similar results are observed in Mo3d XPS spectra of other Ru-MoS_2-x_-CoS_2_/CC samples (Figure 6B), yielding the related data about negative shifts of 0.12 eV for Ru-MoS_2-x_-CoS_2_/CC-240, 0.45 eV for Ru-MoS_2-x_-CoS_2_/CC-280, and 0.50 eV for Ru-MoS_2-x_-CoS_2_/CC-320. It is seen that such an electron injection effect increases gradually with the increase in *m*_Co_, which means more S^2-^ escaping from MoS_2-x_ nanosheets (Liu et al., 2017; Gan et al., 2018) at higher *m*_Co_. Therefore, the atom ratios of S to Mo for all Ru-MoS_2-x_-CoS_2_/CC samples decrease (Supplementary Table S3), and *C*_S-vacancy_ increases (Figure 6E) with increasing *m*_Co_. For instance, Ru-MoS_2-x_-CoS_2_/CC-320 demonstrate the highest *C*_S-vacancy_ (about 27.5%) among all samples.

Additionally, Figure 6 also demonstrates the influence of compositing with CoS_2_ on *C*_S-vacancy_ of the as-synthesized samples without Ru dopants. Figure 6C shows XRD patterns of MoS_2_-CoS_2_/CC-80, MoS_2_-CoS_2_/CC-240, MoS_2_-CoS_2_/CC-280, and MoS_2_-CoS_2_/CC-320. The influences of *m*_Co_ on intensities of the related peaks and RC of MoS_2_ and CoS_2_ are shown in Figure 6C and Supplementary Figure S12, suggesting a decline in the crystallinity of MoS_2_ and an increase in crystallinity of CoS_2_ as well. The trend in the variation of atom ratios of Co to (Co + Mo) of the as-synthesized samples without Ru dopants is the same as that of Ru-doped samples in Figure 6D, which implies the formation of more CoS_2_ and richer heterojunctions at higher *m*_Co_. Similarly, the more heterojunctions are, the stronger electron injection effect from CoS_2_ to MoS_2-x_ is obtained. For instance, Mo 3 day peaks of MoS_2_-CoS_2_/CC-240 display a negative shift of 0.15 eV in comparison with those of MoS_2_-CoS_2_/CC-80 from Supplementary Figure S13. At *m*_Co_ increasing to 320 mg, Mo 3 day peaks of MoS_2_-CoS_2_/CC-320 present a negative shift of about 0.36 eV compared to those of MoS_2_-CoS_2_/CC-80 (Supplementary Figure S13). Therefore, the atom ratios of S to Mo for these samples without Ru dopants also decrease with increasing *m*_Co_ (Supplementary Table S3), leading to an increase in *C*_S-vacancy_ (Figure 6E) as well. Therefore, *C*_S-vacancy_ of the basal planes of Ru-MoS_2-x_ can be efficiently regulated by simply changing *m*_Co_ based on the effect of electron injection from Ru-CoS_2_ to Ru-MoS_2-x_.

Together, either doping Ru or compositing with CoS_2_ only regulates *C*_S-vacancy_ of the as-synthesized samples within a narrow range, such as between 2.1 and 10.5% for doping Ru (Figure 3E) and 4.5 and 11.5% for compositing with CoS_2_ (Figure 6E). Interestingly, synergistically regulating *C*_S-vacancy_ of the as-synthesized samples, from 2.1 to 27.5% (Figure 6E), is realized by a new one-step doping-assisted compositing strategy.

### HER Activities of Ru-MoS_2-X_-CoS_2_/CC Samples Synthesized at Various *V*

**FIGURE 7:**
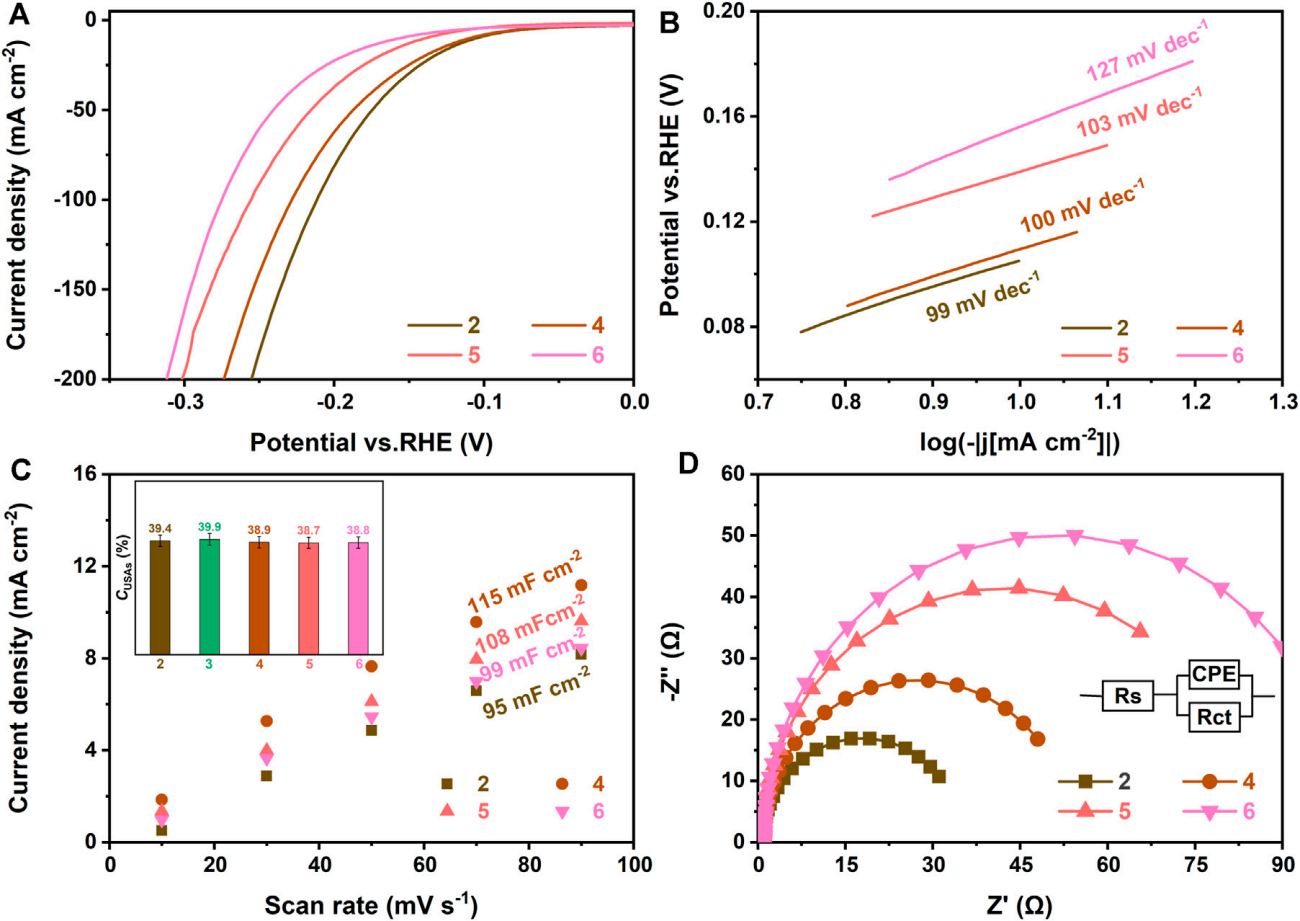
**(A)** Polarization curves, **(B)** Tafel plots, **(C)** *C*_dl_ at different scan rates, and **(D)** Nyquist plots of Ru-MoS_2-x_-CoS_2_/CC-1.0 (2), Ru-MoS_2-x_-CoS_2_/CC-7.0 (4), Ru-MoS_2-x_-CoS_2_/CC-10.0 (5), and Ru-MoS_2-x_-CoS_2_/CC-30.0 (6). The inset of panel **(C)** is *C*_USAs_ of the related samples. The electrolyte is N_2_-saturated 1 M KOH.

**FIGURE 8:**
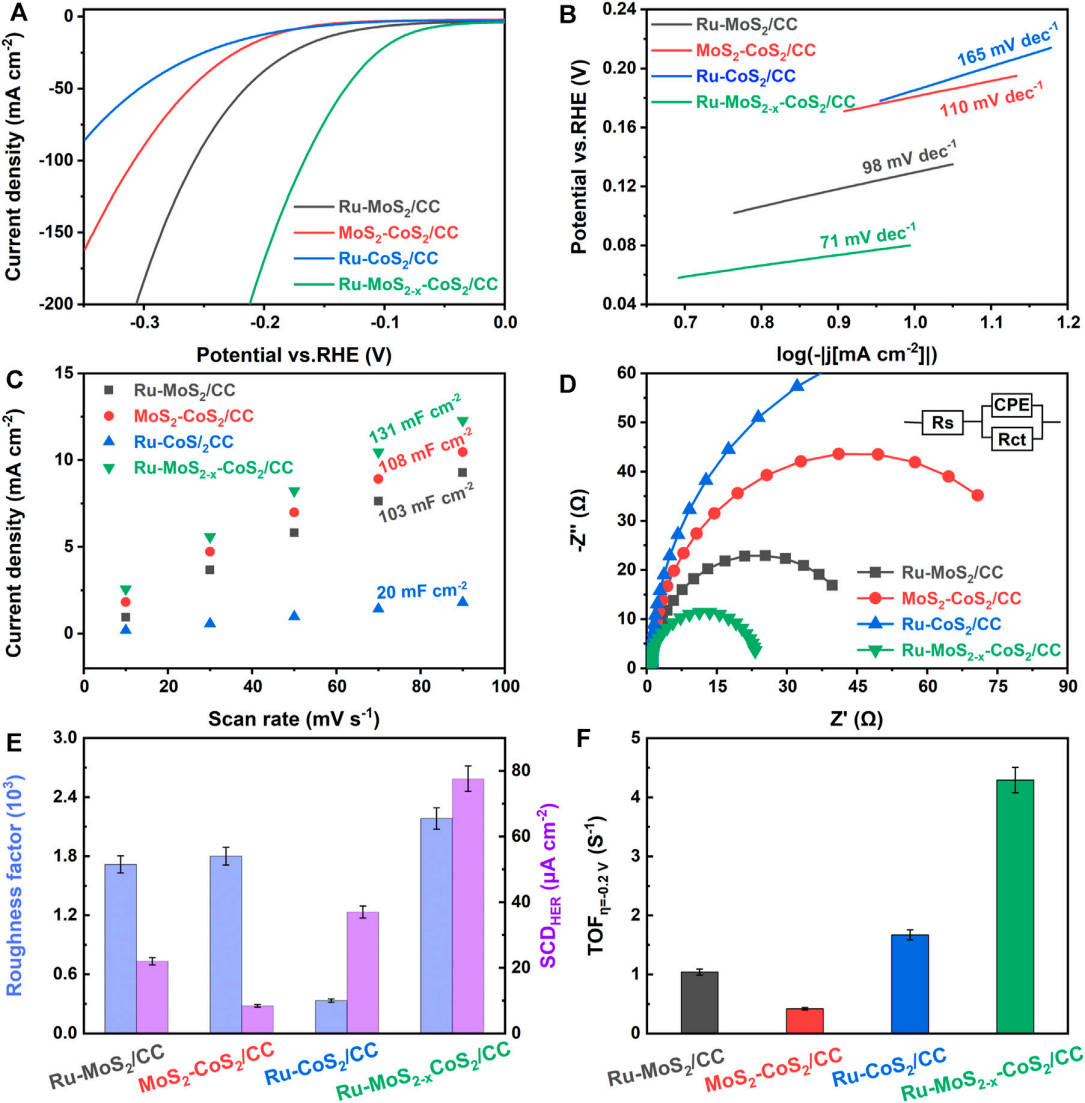
**(A)** Polarization curves, **(B)** Tafel plots, **(C)** *C*_dl_ at different scan rates, **(D)** Nyquist plots, **(E)** *R*_f_ and SCD_HER_, and **(F)** TOF at 0.2 V of Ru-MoS_2_/CC, MoS_2_-CoS_2_/CC, Ru-CoS_2_/CC, and the typical samles. The electrolyte is N_2_-saturated 1 M KOH.

To find out the relationship between the microstructure and HER activity, we carefully investigate a series of electrochemical performances of Ru-MoS_2-x_-CoS_2_/CC samples synthesized at various *V* under the fixed *m*_Co_ (160 mg) in Figures 7, 8. According to Figures 7A–D, the typical samples exhibit the highest HER activity among these samples, such as an overpotential of about 170 mV at a current density of 100 mA cm^−2^ and a Tafel plot of 71 mV dec^−1^ in alkaline environments (1 M KOH), implying that low or excessive Ru dopant is disadvantageous for the HER activity of Ru-MoS_2-x_-CoS_2_/CC samples.

Figure 7C displays double-layer capacitance (*C*_dl_) to evaluate electrochemical active surface area (ECSA), which is calculated by cyclic voltammetry (CV) curves in Supplementary S14, 15. From Figure 7C and Figure 8C, the typical samples also demonstrate the highest *C*_dl_ among all Ru-MoS_2-x_-CoS_2_/CC samples, reaching up to *ca*. 131 mF cm^−2^. For 2H-MoS_2_, its active sites are mainly from unsaturated sulfur atoms (Kibsgaard et al., 2012; Xie et al., 2013a; Liu et al., 2018). This is because the adsorption free energy of hydrogen atom (ΔG_H_) of unsaturated sulfur atoms approaches zero (about -0.06 eV), which indicates their excellent H* adsorption–desorption property (Hinnemann et al., 2005). More recently, modulating sulfur vacancy into MoS_2_-based materials has been developed as an efficient strategy (Tsai et al., 2017; Park et al., 2018; Li Y. et al., 2021) to activate inert basal planes because EMAs can be tailored into newborn active sites (Wang X. et al., 2020). Therefore, the active sites of these Ru-MoS_2-x_-CoS_2_/CC samples should include unsaturated sulfur atoms and EMAs.

To confirm the difference in active sites for such Ru-MoS_2-x_-CoS_2_/CC samples, the inset of Figure 7C further shows the concentration of unsaturated sulfur atoms (USAs) of all samples based on XPS data from Supplementary Figure S16. From this inset, there is no significant difference in the concentration of USAs (*C*_USAs_) for these samples. This may be closely related to preferentially exposed sulfur-edge atoms of Ru-MoS_2-x_ nanosheet array vertically assembled on CC. At the same time, this inset implies that USAs are not the essential factor for the most abundant active sites of the typical samples. From Figure 3E, *C*_S-vacancy_ for Ru-MoS_2-x_-CoS_2_/CC-1.0, the typical samples, Ru-MoS_2-x_-CoS_2_/CC-7.0, Ru-MoS_2-x_-CoS_2_/CC-10.0, and Ru-MoS_2-x_-CoS_2_/CC-30.0 are 7.9, 17.1, 14.0, 12.5, and 12.0%, respectively. It is seen that *C*_S-vacancy_ of the typical samples is higher than that of other Ru-MoS_2-x_-CoS_2_/CC samples. Consequently, we can reasonably conclude that the difference in active sites of RCM/CC should be dependent on *C*_S-vacancy_. The formation of one S-vacancy means the occurrence of three EMAs at *C*_S-vacancy_ < 18% (Li et al., 2016a) because S-vacancy uniformly distributes on the basal planes of 2H-MoS_2-x_. Obviously, under this situation, the higher *C*_S-vacancy_ is obtained, the more EMAs or active sites are achieved. At *m*_Co_ = 160 mg, Ru doping firstly favors heterogeneous nucleation and growth of CoS_2_ (Figures 4C, I) besides introducing a certain quantity S-vacancy into the basal planes. For instance, at *V* increasing to 4.0 ml, the typical samples demonstrate higher *C*_S-vacancy_ (Figure 3E) than Ru-MoS_2-x_-CoS_2_/CC-1.0, maybe due to more heterogeneous interfaces between Ru-CoS_2_ and Ru-MoS_2-x_. Nevertheless, further increasing *V* hinders the formation of Ru-CoS_2_ (Figures 4F, I) and leads to a decline in *C*_S-vacancy_. Lower *C*_S-vacancy_ could originate from fewer Schottky heterojunctions and a weaker electron injection effect of Ru-CoS_2_ at excessively high *V*.

As another key factor for HER, the *R*_CT_ of all Ru-MoS_2-x_-CoS_2_/CC samples is investigated in Figure 7D and Figure 8D. These figures exhibit their Nyquist plots. Each semicircle represents the *R*_CT_ of the cathode reaction. The charge transfer of Ru-MoS_2-x_-CoS_2_/CC-1.0 or Ru-MoS_2-x_-CoS_2_/CC-7.0 is inferior to that of the typical samples. Moreover, further increasing *V* results in considerably unsatisfactory *R*_CT_ from Figure 7D. In our viewpoint, rich active sites will promote the occurrence of HER reaction in the cathode, implying efficient charge transfer for the typical samples. On the other hand, compositing with the metallic phase for fast electrode kinetics (He et al., 2020; Li et al., 2020; Zhou G. et al., 2021), such as CoS_2_ (Li et al., 2020), carbon nanotube (Huang et al., 2017), and reduced graphene oxide (Wang Y. et al., 2020), is one of the important approaches to improving electrocatalytic HER activity. From Figure 7D and Figure 8D, Ru-MoS_2-x_-CoS_2_/CC-1.0 demonstrate better charge transfer compared to Ru-MoS_2_/CC. Furthermore, the influence of *V* on *R*_CT_ is coincident with the trend in the variation of RC of Ru-CoS_2_ (Figure 3C) and atom ratios of Co to (Co + Mo) (inset of Figure 3A) of all Ru-MoS_2-x_-CoS_2_/CC samples. Therefore, the higher crystallinity of cobalt disulfide or the more CoS_2_ is obtained, the lower *R*_CT_ of the as-synthesized samples is achieved.

### HER Activities of Ru-MoS_2-X_-CoS_2_/CC Samples Synthesized at Various *m*_Co_

We further investigate the HER activity of Ru-MoS_2-x_-CoS_2_/CC samples synthesized at various *m*_Co_ under the fixed *V* (4.0 ml) in Supplementary Figure S17. From Supplementary Figures S17A, B, HER activities of Ru-MoS_2-x_-CoS_2_/CC-80 are an overpotential of about 228 mV at a current density of 100 mA cm^−2^ and a Tafel plot of 102 mV dec^−1^. At *m*_Co_ increasing to 160 mg, the typical samples demonstrate higher HER activity than other samples (Figure 8A; Supplementary Figure S17A). Further increasing *m*_Co_ leads to unsatisfactory overpotential and sluggish electrode kinetics, yielding the related data of 264 and 123 mV dec^−1^ for Ru-MoS_2-x_-CoS_2_/CC-240, 294 and 130 mV dec^−1^ for Ru-MoS_2-x_-CoS_2_/CC-280, and 301 and 140 mV dec^−1^ for Ru-MoS_2-x_-CoS_2_/CC-320 at the same current density.

To illustrate the reason why excessive *m*_Co_ is disadvantageous for HER activity, *C*_dl_ and *C*_USAs_ of all samples are tested in Supplementary FIgure S17C and Supplementary Figure S18, respectively. The corresponding CV curves related to Supplementary FIgure S17C are shown in Supplementary Figure S19. From Supplementary FIgure S17C, *C*_dl_ gradually increases with an increase in *m*_Co_. For example, Ru-MoS_2-x_-CoS_2_/CC-80 display low *C*_dl_ (about 118 mF cm^−2^) at *m*_Co_ = 80 mg. At *m*_Co_ increasing to 320 mg, the *C*_dl_ of Ru-MoS_2-x_-CoS_2_/CC-320 is as high as 199 mF cm^−2^. We rationally hypothesize that this is closely related to *C*_S-vacancy_, due to the fact that high *C*_S-vacancy_ favors abundant EMAs. As we know, the basal planes in 2H-MoS_2_ are inert, owing to ΔG_H_ reaching 2.1 eV (Zhou W. et al., 2021). Impressively, inert basal planes can be efficiently activated by a low concentration of S-vacancy (*ca* 4%) modulating into them because the corresponding ΔG_H_ closes to zero (Wang X. et al., 2020). It is not difficult to understand that such EMAs should be highly active, but the number is considerably poor.

According to the related literature (Wang X. et al., 2020), ΔG_H_ negatively shifts with an increase in *C*_S-vacancy_, implying that hydrogen atoms do not easily desorb at excessive *C*_S-vacancy_. For instance, at excessive *m*_Co_, *C*_S-vacancy_ of Ru-MoS_2-x_-CoS_2_/CC-320 reaches up to 27.5%. As expected, EMAs are abundant in this case, but the catalytic sites are of low activity, owing to the undesirable ΔG_H_ (Wang X. et al., 2020). Subsequently, a balance between the intrinsic activity and the number of EMAs to boost highly active EMAs could be realized by precisely regulating *C*_S-vacancy_ to 17.1% (Wang X. et al., 2020). Consequently, the typical samples demonstrate the optimal HER activity among all samples. Furthermore, *C*_USAs_ of all samples except for Ru-MoS_2-x_-CoS_2_/CC-320 is almost the same as that of the typical samples. *C*_USAs_ of Ru-MoS_2-x_-CoS_2_/CC-320 (about 33.9%) is lower than that of other samples, which is responsible for the incompact Ru-MoS_2-x_ shells (Supplementary Figure S11D), and the significant decrease in the content of molybdenum disulfide (Figure 6D). This indicates that sulfur-edge atoms of Ru-MoS_2-x_ nanosheets are not easy to be preferentially exposed for such samples synthesized at excessive *m*_Co_.

Last but not least, Supplementary Figure S17Dand Figure 8D further exhibit *R*_CT_ of all samples. Higher *m*_Co_ leads to higher *R*_CT_ of the related samples (Supplementary Figure S17D). For example, at *m*_Co_ reaching up to 320 mg, Ru-MoS_2-x_-CoS_2_/CC-320 display the highest *R*_CT_ among all Ru-MoS_2-x_-CoS_2_/CC samples. It can be ascribed to insufficient highly active EMAs regardless of the higher RC of Ru-CoS_2_ (Figure 6A; Supplementary Figure S10) for these samples synthesized at higher *m*_Co_. Insufficient active sites could not promote redox half-reaction, thereby leading to inefficient charge transfer.

### Comparison of HER Activities of the Typical Samples and Other Similar Electrocatalysts

HER activities of Ru-MoS_2_/CC, MoS_2_-CoS_2_/CC, Ru-CoS_2_/CC, and the typical samples are shown in Figure 8A. Ru-CoS_2_/CC display the lowest HER activity among these samples, suggesting that the activity of the typical samples is mainly from Ru-MoS_2-x_ rather than Ru-CoS_2_. In addition, Ru-MoS_2_/CC exhibit faster electrode kinetics than MoS_2_-CoS_2_/CC according to Figure 8B. In our viewpoint, the Ru-doped sites can significantly accelerate the sluggish water dissociation in alkaline HER (Li J. et al., 2021).

From Figure 8C, *C*_dl_ of Ru-MoS_2_/CC is only 103 mF cm^−2^ and lower than that of the typical samples. Furthermore, *C*_USAs_ of Ru-MoS_2_/CC (about 39.3%) is almost the same as that of the typical samples from Supplementary Figure S18. Therefore, the difference in active sites between Ru-MoS_2_/CC and the typical samples should be closely related to *C*_S-vacancy_. Higher *C*_S-vacancy_ indicates more highly active EMAs. As shown in Figure 3E, *C*_S-vacancy_ of Ru-MoS_2_/CC is only about 3.1% by doping Ru, implying insufficient active sites. In our strategy, doping Ru coupled to compositing with CoS_2_ synergistically regulates *C*_S-vacancy_ of the as-synthesized samples from 2.1 to 27.5%. For instance, *C*_S-vacancy_ of the typical samples reaches up to 17.1% (Figure 3E), which implies that rich highly active sites are successfully introduced into the typical samples. In terms of Figure 8D, *R*_CT_ of the typical samples is lower than that of Ru-MoS_2_/CC. Besides rich active sites promoting redox half-reaction, high crystallized Ru-CoS_2_ of the typical samples is another important factor to realize the efficient charge transfer during electrocatalysis. Next, the specific activity and turnover frequency (TOF) are further conducted to investigate the intrinsic activity of all samples (Figures 8E, F). *C*_dl_ of an ideal plane electrode is considered as 60 μF cm^−2^ (Levine and Smith, 1971); the roughness factor (*R*_f_) can be calculated by the formula: *R*_f_ = (C_dl_ 60^-1^)×10^+3^. The specific alkaline HER current density (SCD_HER_) is determined by the formula: SCD_HER_ = *j R*_f_^−1^ (Jovic et al., 2015; Jovic et al., 2016), where *j* is the current density at an overpotential of -0.2 V. From Figure 8E, the typical samples have not only the largest *R*_f_ (2.18 × 10^+3^) but also the highest specific activity (about 77.6 μA cm^−2^) among all samples. In addition, the TOF for these samples is obtained by the formula TOF = *jS* (2n*F*) ^−1^. Here, *S* is the geometrical surface area in cm^2^ and *F* is the Faraday constant. The value of n is the number of active sites (mol), which is confirmed according to the previously reported literature (Tian et al., 2014). The typical samples also exhibit the highest intrinsic activity among all samples, about 4.29 s^−1^ in Figure 8F. Moreover, the comparison between the typical samples and other similar electrocatalysts is provided in Table 1. The typical samples exhibit higher HER activity than other similar electrocatalysts listed in Table 1, which is ascribed to two factors: abundant active sites and accelerated electrode kinetics during the HER process.

**TABLE 1:** Comparison of the HER electrocatalytic activity of Ru-MoS_2-x_-CoS_2_/CC with some MoS_2_-based HER electrocatalysts recently reported.

Samples	η_10_ (mV)	Ref
Co_3_S_4_/MoS_2_ NRs	−166	Li et al. (2022b)
MoS_2_@CoSe_2_-CC	−101	Yuan et al. (2022)
NiS_2_/MoS_2_@GNS	−130	Balaji et al. (2022)
N-doped MoS_2_/Ti_3_C_2_T_x_	−80	Liu et al. (2021)
Co-E_x_-MoS_2_	−89	Luo et al. (2018)
SA-Ru-MoS_2_	−76	Zhang et al. (2019a)
0.2NM (Ni(OH)_2_/MoS_2_)	−227	Zhao et al. (2018)
Co_9_S_8_-MoS_2_@3DC	−177	Diao et al. (2019)
Ru-MoS_2_	−98	Geng et al. (2022)
ZnS@C@MoS_2_	−118	Liu et al. (2019)
Ru-MoS_2-x_-CoS_2_/CC	−73	This work

### The Long-Term Durability of the Typical Samples

In addition, LSV curves of the typical samples before and after 1000 cycles are recorded to evaluate their durability, and just a 10 mV negative shift at a current density of -100 mA cm^−2^ is seen in Supplementary Figure S20A, B; the chronoamperometric response (i ∼ t) of the typical samples displays negligible attenuation of the current density for 10 h. Moreover, no significant change is observed in the morphology and phase structure after 1000 cycles (Supplementary Figures S21, 22). All results suggest that the typical samples possess remarkable long-term durability for HER in alkaline media.

## Conclusion

Herein, we develop a one-step Ru doping coupled to compositing with the CoS_2_ strategy for the fabrication of Ru-MoS_2-x_-CoS_2_/CC. In our strategy, Ru doping is advantageous for the formation of S-vacancy in the basal planes of MoS_2_. More importantly, Ru doping affects microstructures of CoS_2_, which has a significant influence on *C*_S-vacancy_ of Ru-MoS_2-x_ nanosheets in Ru-MoS_2-x_-CoS_2_/CC samples in turn. At the fixed *m*_Co_ (160 mg), Ru doping favors the heterogeneous nucleation and growth of CoS_2_ at *V* increasing to 4.0 ml, which leads to a high crystallinity of Ru-CoS_2_ and rich heterogeneous interfaces between Ru-CoS_2_ and Ru-MoS_2-x_. This facilitates the electron transfer from Ru-CoS_2_ to Ru-MoS_2-x_, indicating an increase in *C*_S-vacancy_, thereby increasing *C*_S-vacancy_ of the MoS_2_-based materials. However, further increasing *V* results in a low crystallinity of Ru-CoS_2_ and poor heterojunctions, implying a weaker electron injection effect of Ru-CoS_2_ and low *C*_S-vacancy_. At fixed *V* (4.0 ml of RuCl_3_ solution), the electron injection effect increases gradually with the increase in *m*_Co_, which means more S^2-^ escaping from Ru-MoS_2_ nanosheets at higher *m*_Co_. Therefore, synergistically regulating *C*_S-vacancy_ of the as-synthesized samples, from 2.1 to 27.5%, is realized by a new one-step Ru doping coupled to compositing with the CoS_2_ strategy. High *C*_S-vacancy_ indicates abundant EMAs. Impressively, inert basal planes can be efficiently activated by modulating low *C*_S-vacancy_ into Ru-MoS_2-x_-CoS_2_/CC samples. However, the number is considerably poor. Additionally, hydrogen atoms do not easily desorb at excessive *C*_S-vacancy_. This is because EMAs are abundant in this case, but the catalytic sites are of low activity, owing to the undesirable ΔG_H_. By precisely regulating *C*_S-vacancy_ to 17.1%, a balance between the intrinsic activity and the number of EMAs to boost highly active EMAs should be realized. Consequently, the typical samples demonstrate the optimal alkaline HER activity among all samples, such as a low overpotential of 170 mV at 100 mA cm^−2^, a large SCD_HER_ of 77.6 μA cm^−2^, and a TOF of 4.29 s^−1^ at - 0.2 V as well as excellent long-term stability. The results pave a new approach to activating inert basal planes in MoS_2_ for efficient hydrogen evolution and promise important applications in the fields of electrocatalysis or energy conversion.

## Data Availability

The original contributions presented in the study are included in the article/Supplementary Material; further inquiries can be directed to the corresponding authors.
