# Supplementary material for: Synergistic Regulation of S-Vacancy of MoS2-Based Materials for Highly Efficient Electrocatalytic Hydrogen Evolution
Source: Front Chem. 2022 Jun 8;10:915468. doi: 10.3389/fchem.2022.915468 (PMC9214220; doi:10.3389/fchem.2022.915468)
Supplement: Supplementary file 1 [file Presentation1.pdf]

Supplementary Material

Synergistic regulation of S-vacancy of MoS_2-based materials for highly efficient electrocatalytic hydrogen evolution

Running title: Regulating S-vacancy of MoS_2-based materials for efficient HER

Xiao-yun Li[3], Shao-ju Zhu[2], Yi-long Wang[*, 1], Tian Lian[2], Xiao-yu Yang[2], Cui-fang Ye[4], Yu Li[2], Bao-lian Su[2] and Li-hua Chen[*, 2]

[1] School of Chemistry, Chemical Engineering and Life Science, Wuhan University of Technology, Wuhan, 430070, Hubei, China.

[2] State Key Laboratory of Advanced Technology for Materials Synthesis and Processing, Wuhan University of Technology, Wuhan, 430070, Hubei,

[3] State Key Laboratory of Silicate Materials for Architectures, Wuhan University of Technology, Wuhan, Hubei 430070, China.

[4] Department of Histology and Embryology, Tongji Medical College, Huazhong University of Science and Technology, Wuhan, Hubei 430030, China.

*** Corresponding author: Prof. Yi-long Wang (wangyilong@whut.edu.cn);**

Prof. Li-hua Chen (chenlihua@whut.edu.cn).

Contents

Experimental Section of Supplementary Material 4-6

Figure S1 7

Figure S2 8

Figure S3 9

Figure S4 10

Figure S5 11

Figure S6 12

Figure S7 13

Figure S8 14

Figure S9 15

Figure S10 16

Figure S11 17

Figure S12 18

Figure S13 19

Figure S14 20

Figure S15 21

Figure S16 22

Figure S17 23

Figure S18 24

Figure S19 25

Figure S20 26

Figure S21 27

Figure S22 28

Table S1 29

Table S2 30

Table S3 31

Experimental Section of Supplementary Material

Reagents and materials

Carbon cloth (CC) was purchased from PINE Technology Holdings Co., Ltd., Hongkong, China. $Na_2MoO_4·4H_2O$, CH_4N_2S, $Co(NO_3)_2·6H_2O$, and other chemical reagents were purchased from Sinopharm Chemical Reagent Co., Ltd., China. These chemical reagents were of analytical reagent grade and used without further purification. Deionized water with a resistivity of 18 MΩ·cm was used in our work.

Pretreatment of CC

A piece of CC with a size of 2 × 2 cm^2 was immersed in concentrated nitric acid at room temperature for 30 min and then transferred into a 100 mL Teflon-lined autoclave and heated at 120 ℃ for 30 min. After cooling to room temperature naturally, the pretreated CC was cleaned with deionized water and ethanol several times under ultrasonication for 15 min, respectively. Finally, it was dried under a 60 ℃ oven for further use.

Characterization

The phase structures of all samples were examined by using powder X-ray diffraction (XRD) patterns using a Bruker AXS D8-Advance, a diffractometer with Cu Ka radiation (λ= 0.15418 nm) in the 2θ range from 5 ° to 65 ° at a scanning step of 0.05 °. X-ray photoelectron spectroscopy (XPS) measurements were employed by a VG Multilab 2000 X XPS system equipped with the Al Ka source. All the binding energies

were referenced to the C1s peak at 284.8 eV of the surface adventitious carbon. Transmission electron microscope (TEM) and high-resolution TEM (HR-TEM) images were investigated by a JEOL JEM-2100F HRTEM, and the accelerating voltage was 200 kV. For the preparation of TEM samples, Ru-MoS_2 nanosheets coated Ru-CoS_2 samples must fall off CC by ultrasonic treatment. Before TEM analysis, these samples were prepared by depositing a single drop of diluted sample dispersion in ethanol onto a copper grid coated with an amorphous carbon film. Electron paramagnetic resonance (EPR) spectra were measured using Bruker A330 X-band continuous wave EPR spectrometer. The paramagnetic samples were placed in a rectangular resonate at 9.853301 GHz. Morphologies of all samples were observed by a field emission scanning electron microscope (FESEM; Hitachi S-4800) operated at an accelerating voltage of 5.0 kV. The elemental composition of RCM/CC was also characterized by an energy-dispersive spectrometer (EDX) associated with FESEM. An inductively coupled plasma-optical emission spectroscopy (ICP-OES; Prodigy 7) was used to quantitatively analyze the metal contents of electrocatalysts. Fourier-transforming infrared (FT-IR) absorption spectra were performed with a Nexus spectrometer (Nicolet6700) in the range of 4000 ~ 400 cm^{-1} with 32 scans with the samples in a KBr disk. A micro-Raman study was performed on the Renishaw in Via (Britain) laser confocal Raman microscope at room temperature under an excitation wavelength of 633 nm with He-Ne laser.

Electrochemical measurements

Electrochemical measurements were carried out in a standard three-electrode system on a CHI 660E electrochemical workstation (Shanghai Chenhua Instruments, China) at room temperature with as-obtained samples RCM/CC (area: 0.5 × 0.5 cm^2) as the working electrode, a graphite rod as the counter electrode and a Hg/HgO electrode (1 M KOH solution) as the reference electrode. All experiments were done in the electrolyte of 1 M KOH bubbled with N2. Unless otherwise specified, all potentials in electrochemical measurements were converted to the potentials *vs* the reversible hydrogen electrode (RHE) according to the following Equation 01:

$$\mathrm{E}(vs.\,\mathrm{RHE}) = \mathrm{E}(vs.\,\mathrm{Hg/HgO}) + 0.059 \times \mathrm{pH} + 0.098\mathrm{V} \qquad (01)$$

For HER measurements, line sweep voltammetry (LSV) was measured at a scan rate of 5 mV s^{-1}. Polarization curves were recorded between 0 and – 0.35 V *vs.* RHE. For the investigation of electrochemical double-layer capacitances (C_{dl}), cyclic voltammetry (CV) was measured at the scan rates of 10, 30, 50, 70, and 90 mV s^{-1}, respectively, in the potential ranging from 0 to 0.2 V *vs.* RHE. Chronoamperometric responses (i ~ t) methods were conducted to record the long-term durability. The Nyquist plots were measured with frequencies ranging from 100 kHz to 0.01 Hz at an overpotential of 20 mV *vs.* RHE. The impedance data were fitted to a simplified Randles circuit to extract the series and charge-transfer resistances. All the experiments were carried out without an activation process and done at room temperature.

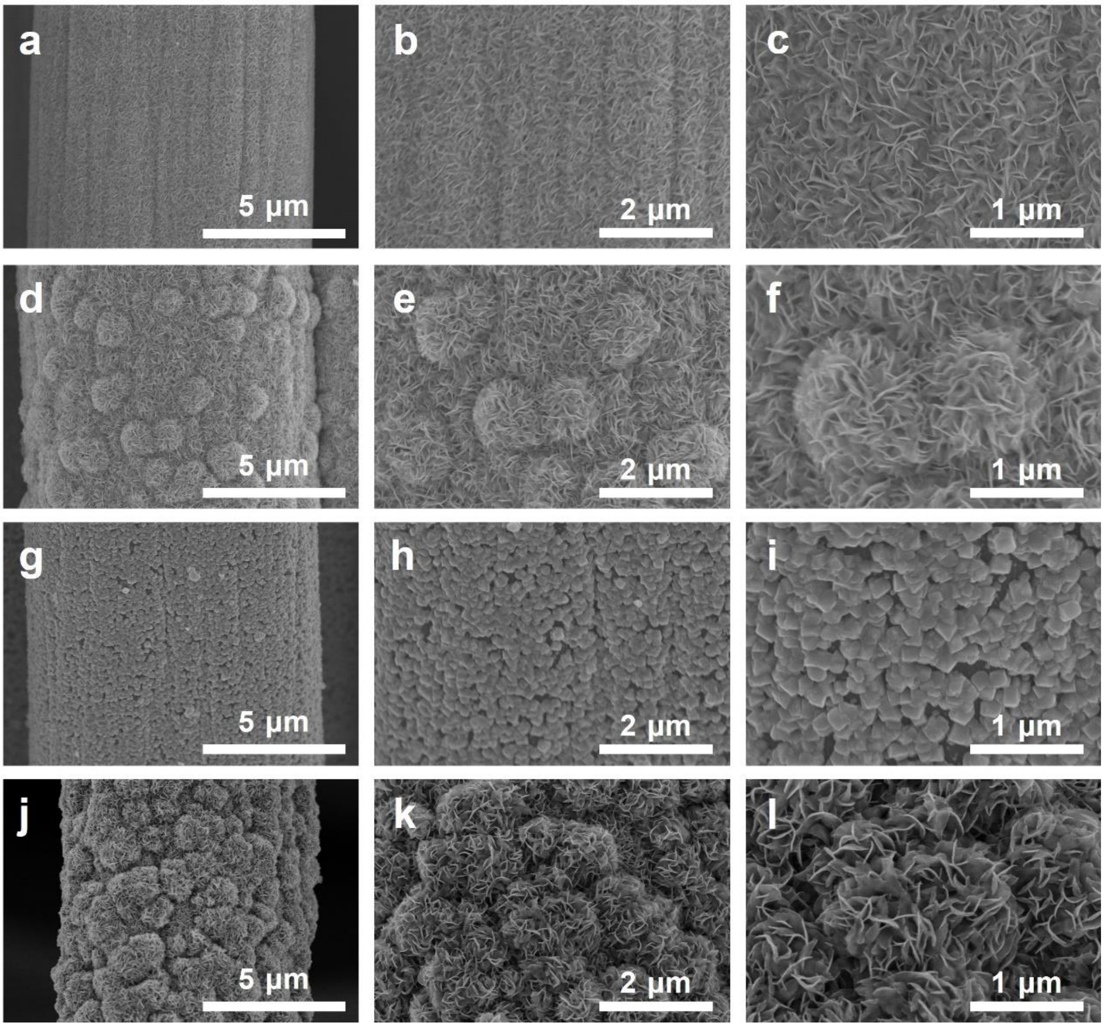

Figure S1 SEM images of (a, b, and c) Ru-MoS_2/CC, (d, e, and f) MoS_2-CoS_2/CC, and (g, h and i) Ru-CoS_2/CC and (j, k, and l) Ru-MoS_{2-x}-CoS_2/CC.

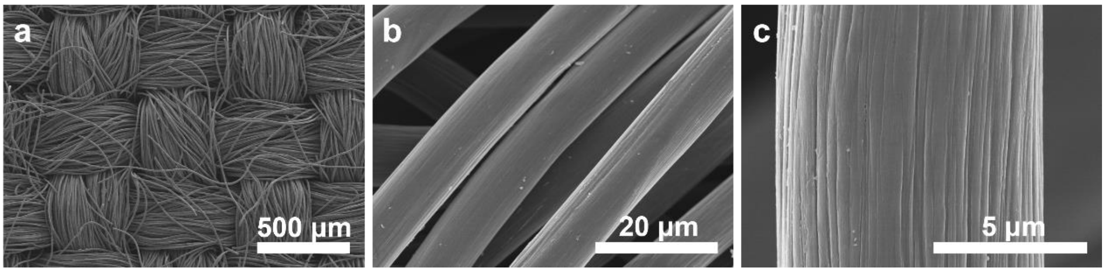

Figure S2 SEM images of CC.

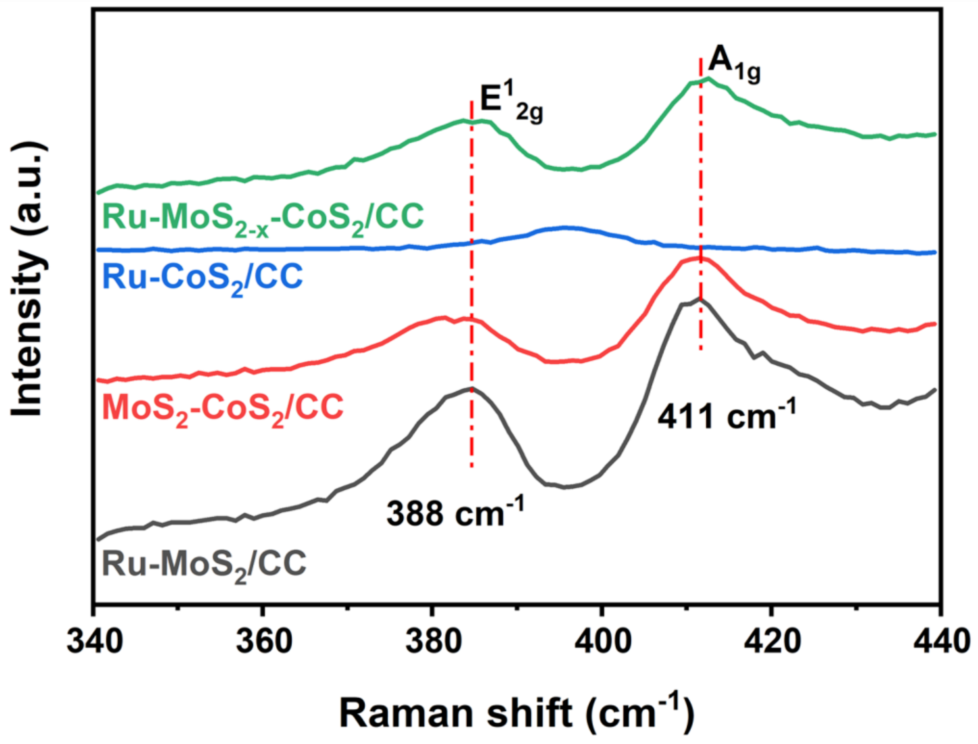

Figure S3 Raman spectra of Ru-MoS_2/CC, MoS_2-CoS_2/CC, Ru-CoS_2/CC, and Ru-MoS_{2-x}-CoS_2/CC.

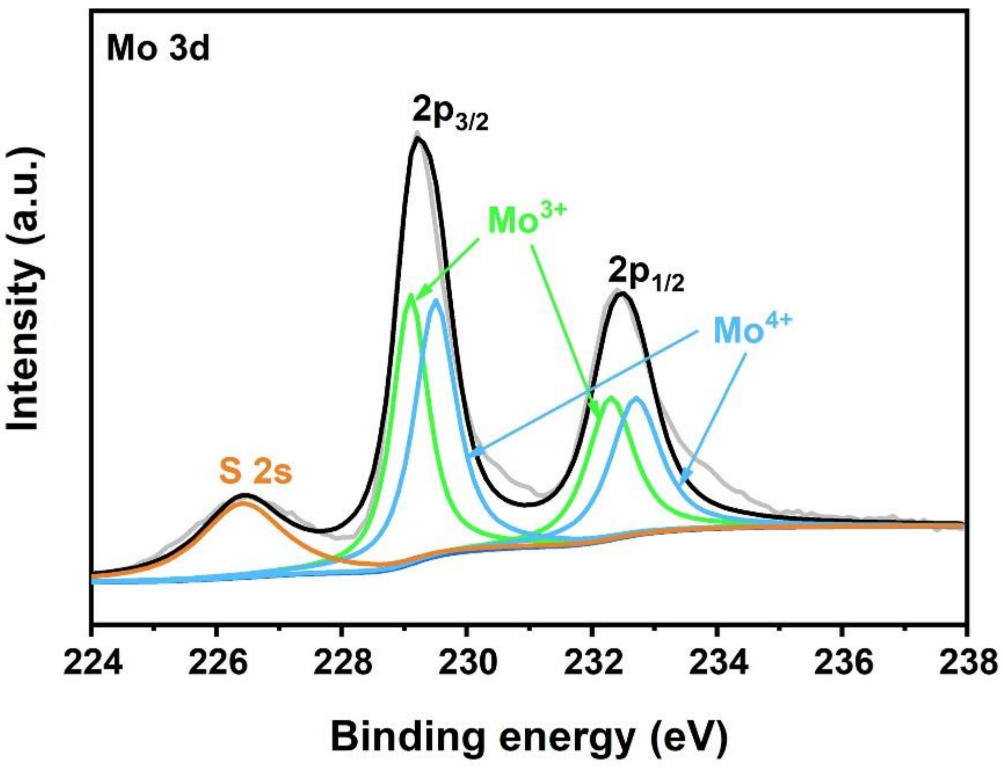

Figure S4 Mo 3d high-resolution XPS spectra of MoS_2/CC

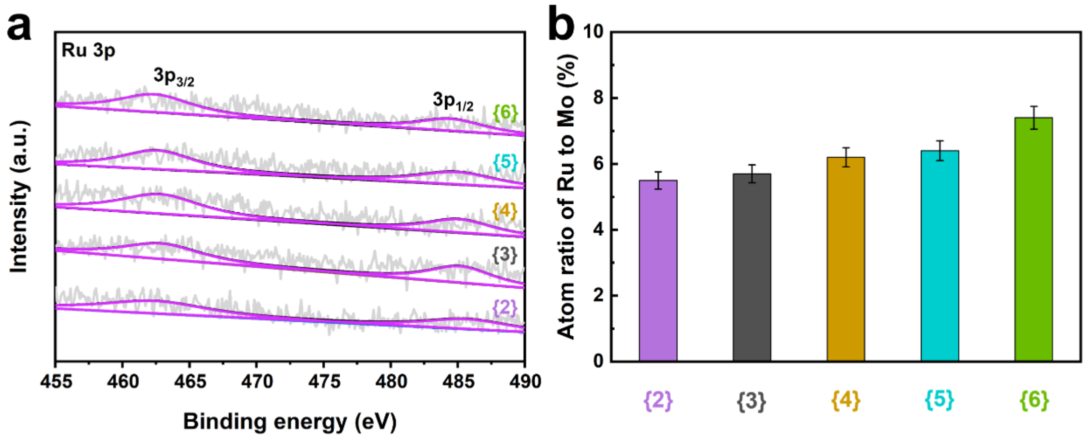

Figure S5 (a) Ru 3p high-resolution XPS spectra and (b) atom ratio of Ru to Mo by XPS data of Ru-MoS_2/CC-1.0 ({2}), Ru-MoS_2/CC ({3}), Ru-MoS_2/CC-7.0 ({4}), Ru-MoS_2/CC-10.0 ({5}) and Ru-MoS_2/CC-30.0 ({6}).

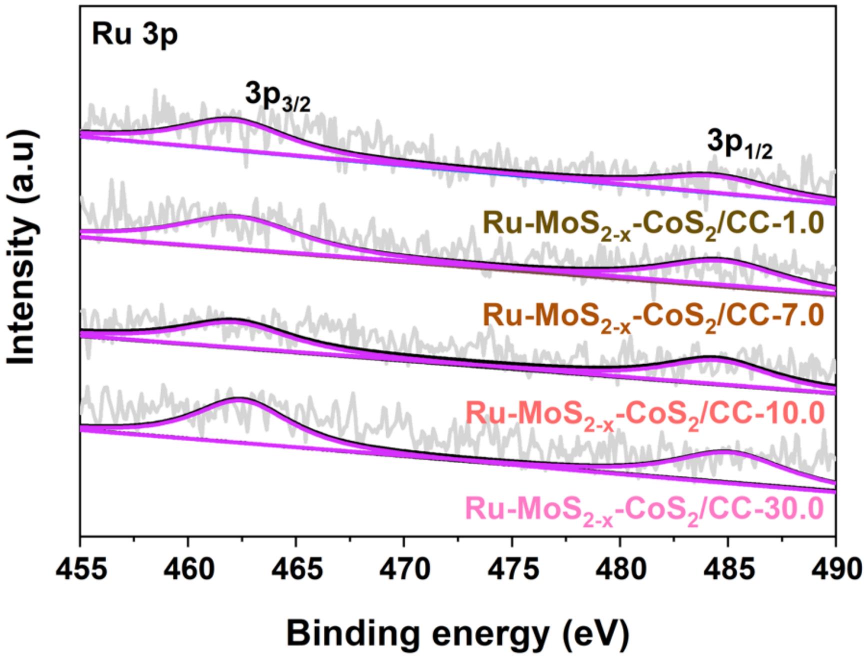

Figure S6 Ru 3p high-resolution XPS spectra of Ru-MoS_{2-x}-CoS_2/CC-1.0, Ru-MoS_{2-x}-CoS_2/CC-7.0, Ru-MoS_{2-x}-CoS_2/CC-10.0 and Ru-MoS_{2-x}-CoS_2/CC-30.0.

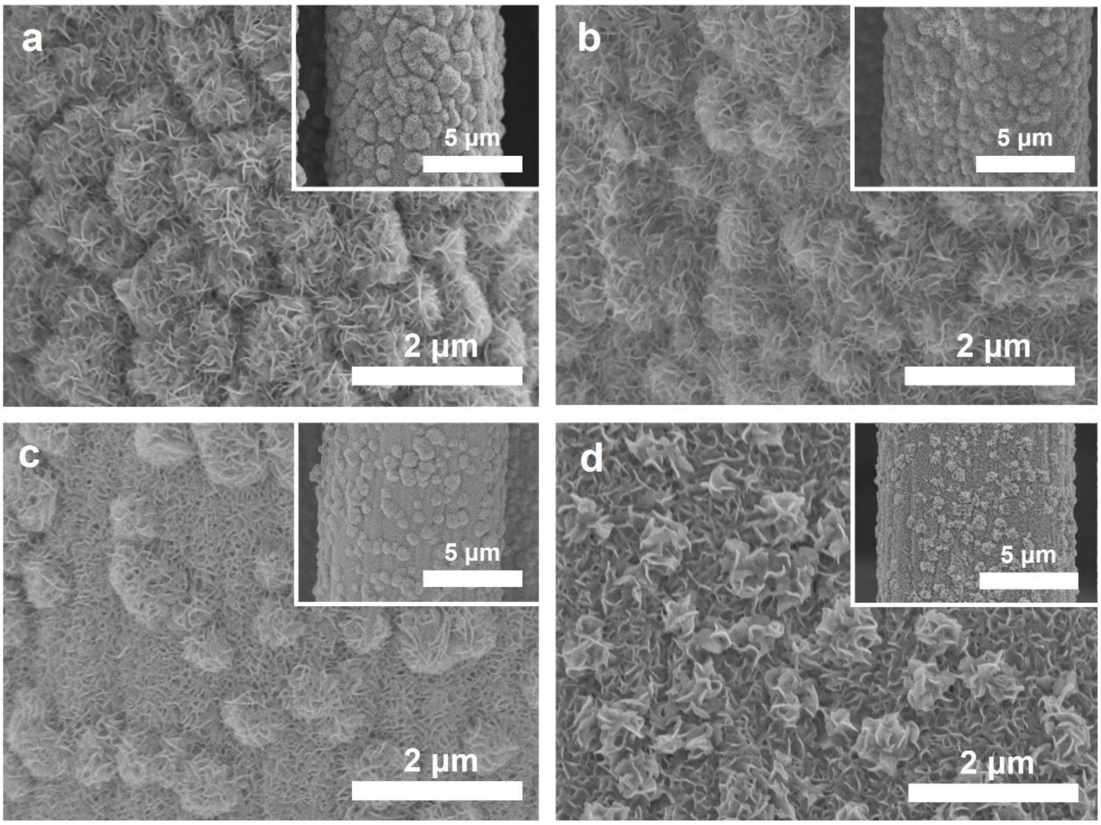

Figure S7 SEM images of (a) Ru-MoS_{2-x}-CoS_2/CC-1.0, (b) Ru-MoS_{2-x}-CoS_2/CC-7.0, (c) Ru-MoS_{2-x}-CoS_2/CC-10.0 and (d) Ru-MoS_{2-x}-CoS_2/CC-30.0. Insets are the low magnified images of the related samples.

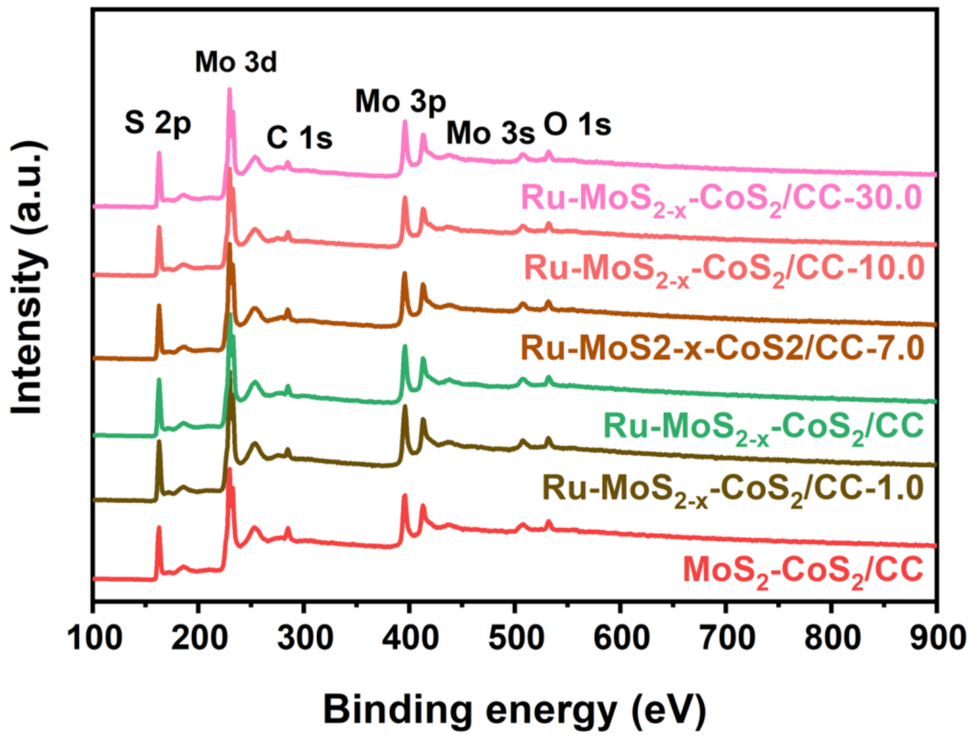

Figure S8 Full XPS spectra of MoS_2-CoS_2/CC, Ru-MoS_{2-x}-CoS_2/CC-1.0, Ru-MoS_{2-x}-CoS_2/CC, Ru-MoS_{2-x}-CoS_2/CC-7.0, Ru-MoS_{2-x}-CoS_2/CC-10.0 and Ru-MoS_{2-x}-CoS_2/CC-30.0.

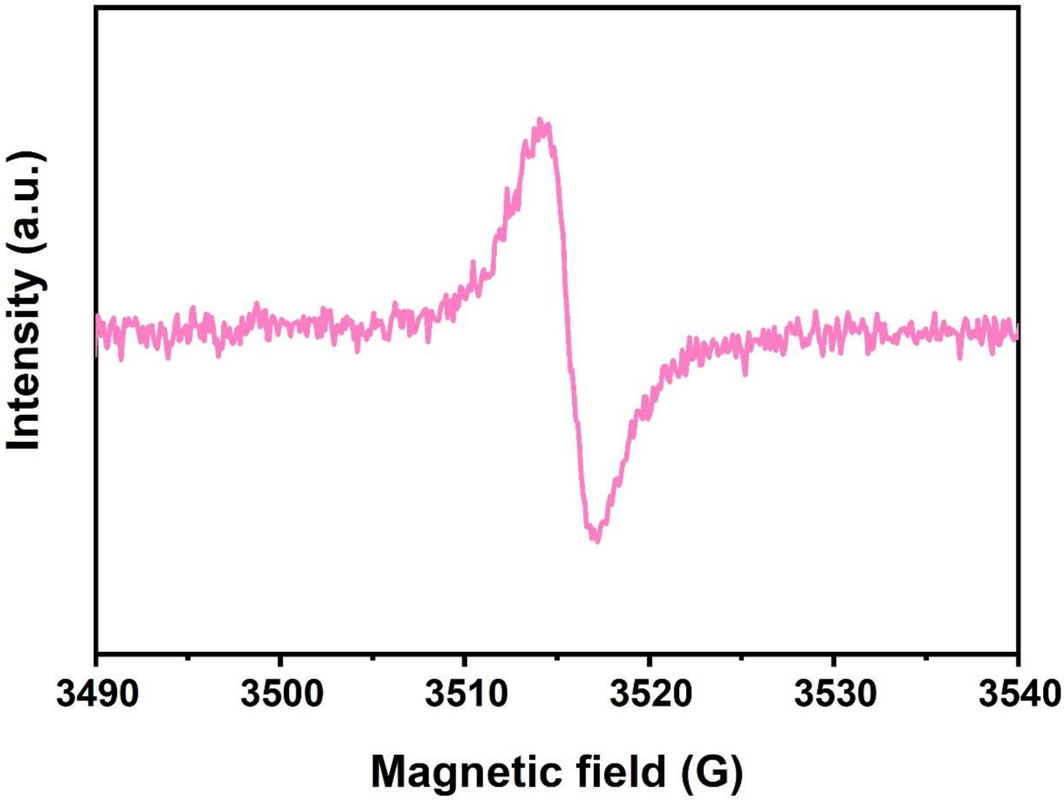

Figure S9 EPR spectrum of Ru-MoS_{2-x}-CoS_2/CC-30.0.

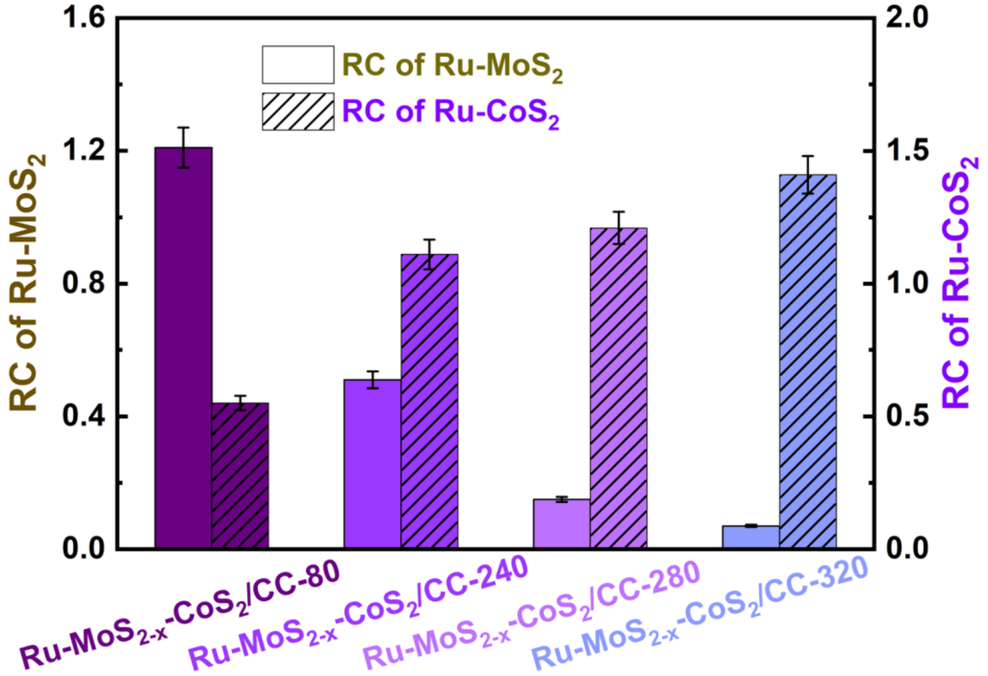

Figure S10 Relative crystallinity (RC) of the related samles based on the corresponding XRD data such as (002) plane for molybdenum disulfide, and (200), (210), (211), (220) and (311) planes for cobalt disulfide. Meanwhile, Ru-MoS_{2-x}-CoS_2/CC-80, Ru-MoS_{2-x}-CoS_2/CC-240, Ru-MoS_{2-x}-CoS_2/CC-280, and Ru-MoS_{2-x}-CoS_2/CC-320 were synthesized at the mass of $Co(NO_3)_2 \cdot 6H_20$ = 80, 240, 280, and 320 mg under otherwise the same conditions of the typical experiments, respectively.

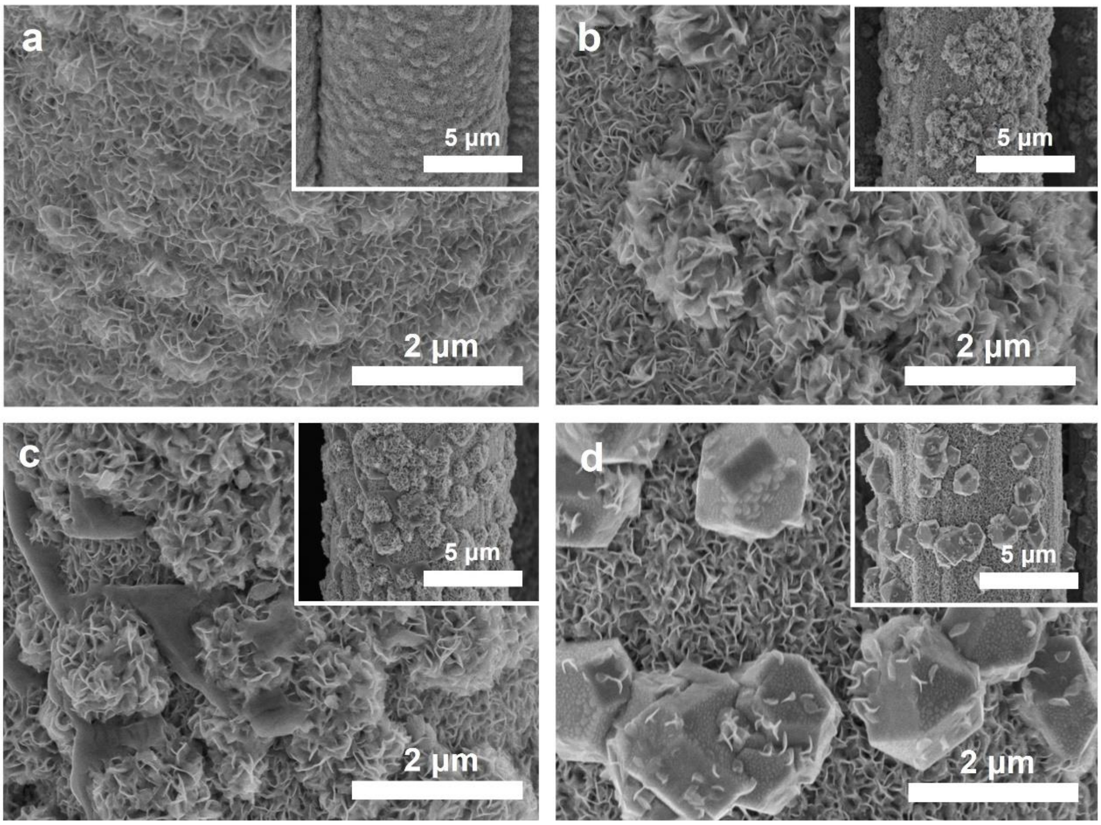

Figure S11 SEM images of (a) Ru-MoS_{2-x}-CoS_2/CC-80, (b) Ru-MoS_{2-x}-CoS_2/CC-240, (c) Ru-MoS_{2-x}-CoS_2/CC-280, and (d) Ru-MoS_{2-x}-CoS_2/CC-320. Insets are the low magnified SEM images of the corresponding samples.

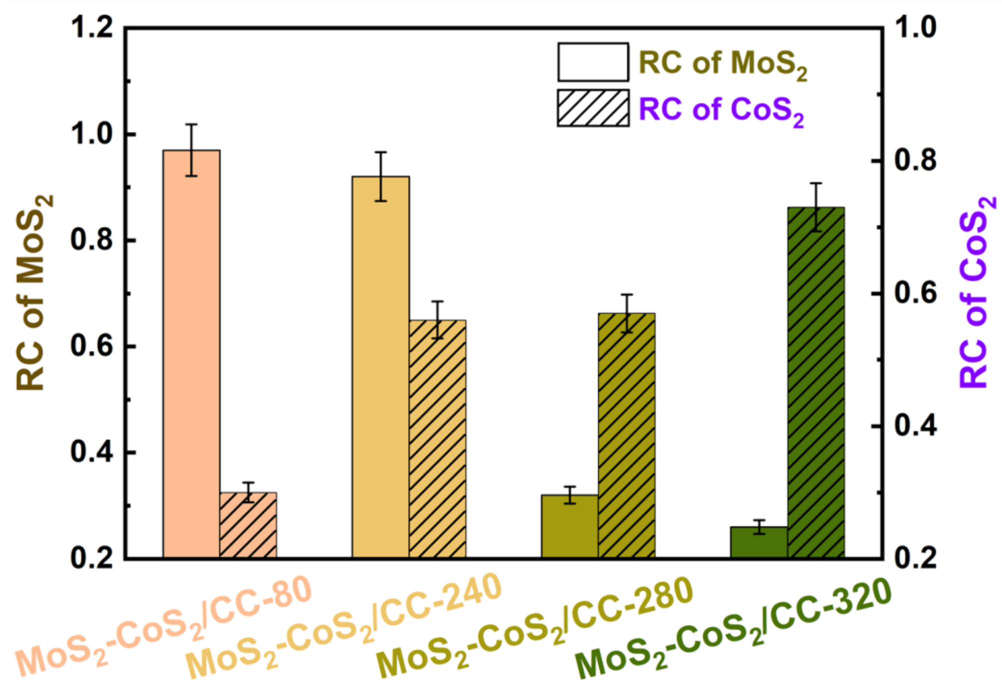

Figure S12 RC of MoS_2 and CoS_2 of the related samples based on the corresponding XRD data such as (002) plane for molybdenum disulfide, and (200), (210), (211), (220) and (311) planes for cobalt disulfide. Meanwhile, fabrications of MoS_2-CoS_2-80, MoS_2-CoS_2-240, MoS_2-CoS_2-280, and MoS_2-CoS_2-320 are almost the same as those of Ru-MoS_{2-x}-CoS_2/CC-80, Ru-MoS_{2-x}-CoS_2/CC-240, Ru-MoS_{2-x}-CoS_2/CC-280, and Ru-MoS_{2-x}-CoS_2/CC-320 except for the absence of $RuCl_3$ solution.

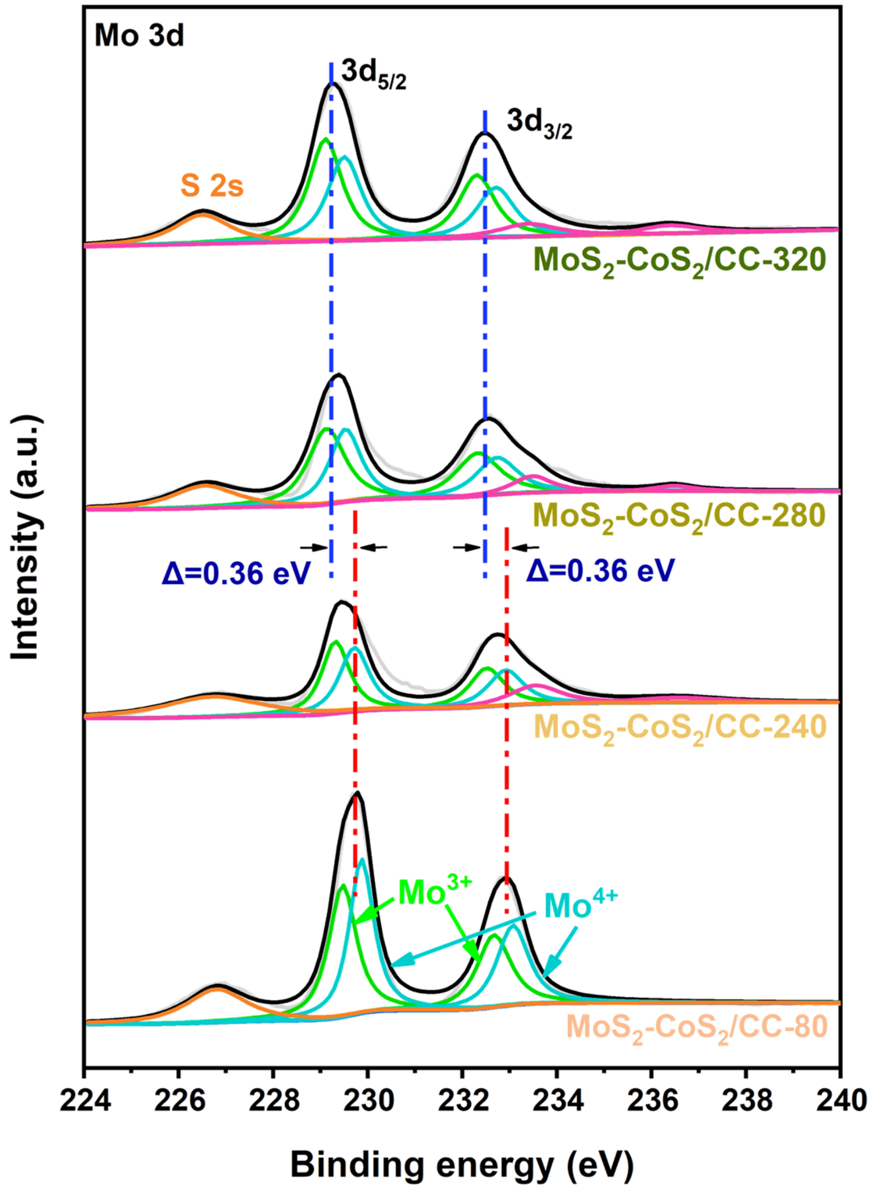

Figure S13 (a) Mo3d high-resolution XPS spectra of MoS_2-CoS_2-80, MoS_2-CoS_2-240, MoS_2-CoS_2-280, and MoS_2-CoS_2-320.

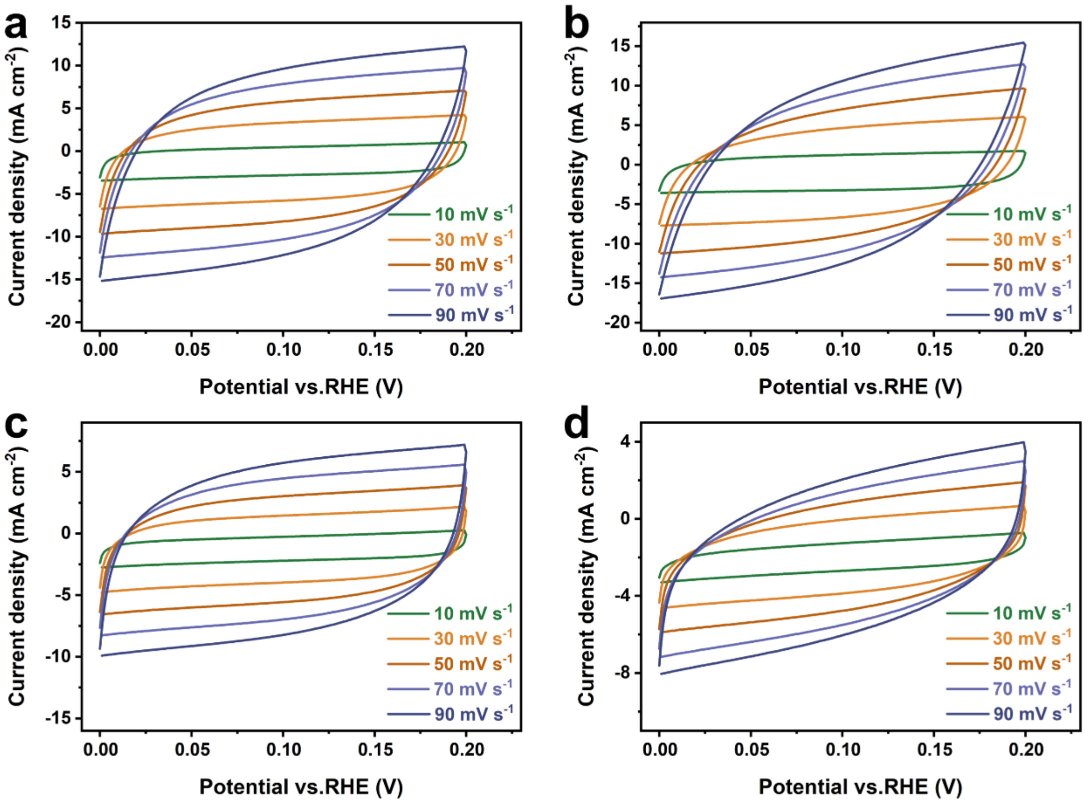

Figure S14 CV curves of (a) Ru-MoS_{2-x}-CoS_2/CC-1.0, (b) Ru-MoS_{2-x}-CoS_2/CC-7.0, (c) Ru-MoS_{2-x}-CoS_2/CC-10.0 and (d) Ru-MoS_{2-x}-CoS_2/CC-30.0. The scan rates are 10, 30, 50, 70 and 90 mV/s, respectively. The electrolyte is N_2-saturated 1 M KOH.

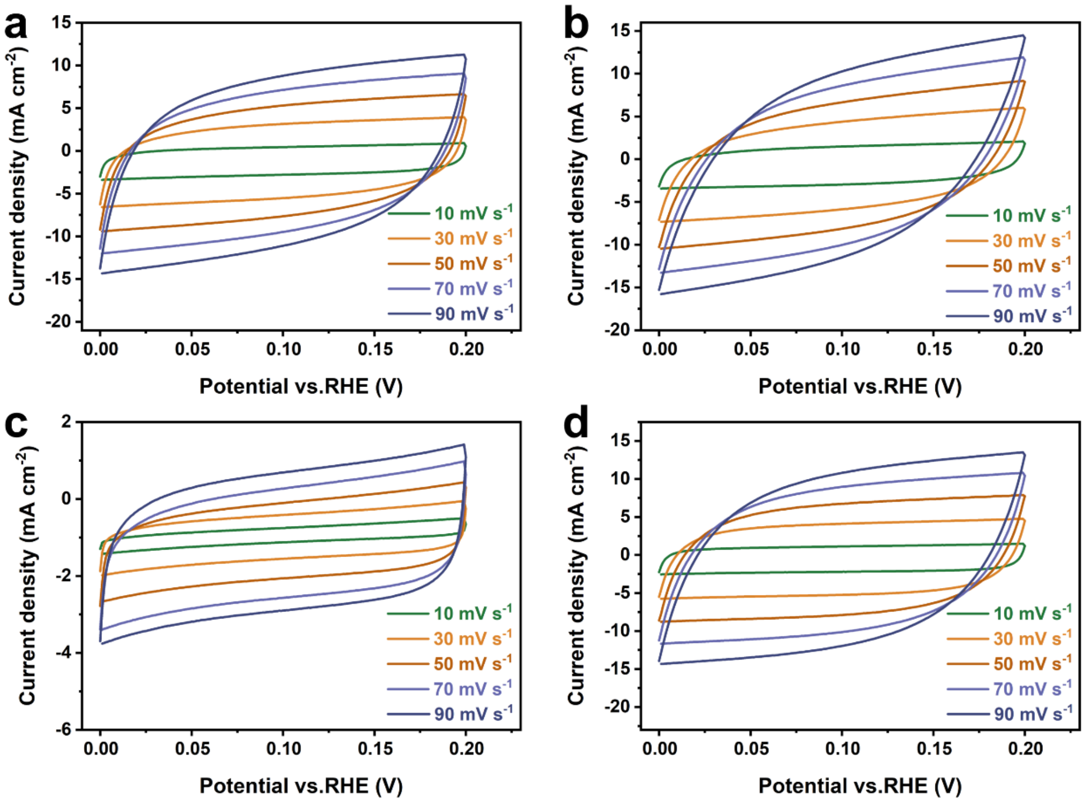

Figure S15 CV curves of (a) Ru-MoS_2/CC, (b) MoS_2-CoS_2/CC, (c) Ru-CoS_2/CC, and (d) Ru-MoS_{2-x}-CoS_2/CC. The scan rates are 10, 30, 50, 70, and 90 mV/s, respectively. The electrolyte is N_2-saturated 1 M KOH.

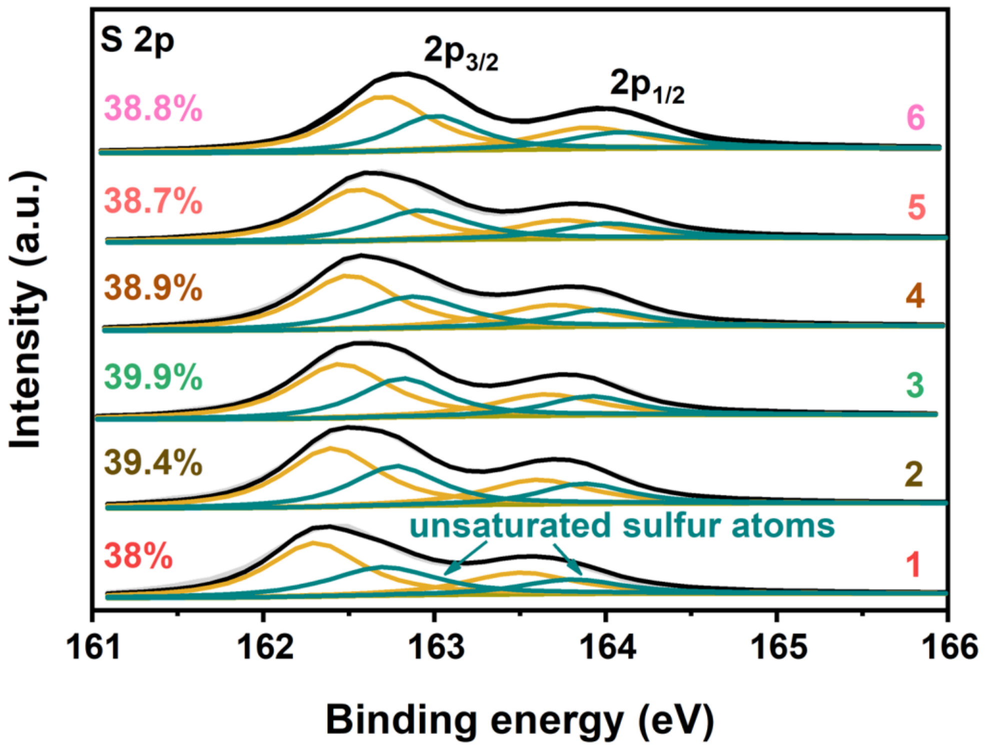

Figure S16 S2p high-resolution XPS spectra of MoS_2-CoS_2/CC (1), Ru-MoS_{2-x}-CoS_2/CC-1.0 (2), Ru-MoS_{2-x}-CoS_2/CC (3), Ru-MoS_{2-x}-CoS_2/CC-7.0 (4), Ru-MoS_{2-x}-CoS_2/CC-10.0 (5) and Ru-MoS_{2-x}-CoS_2/CC-30.0 (6).

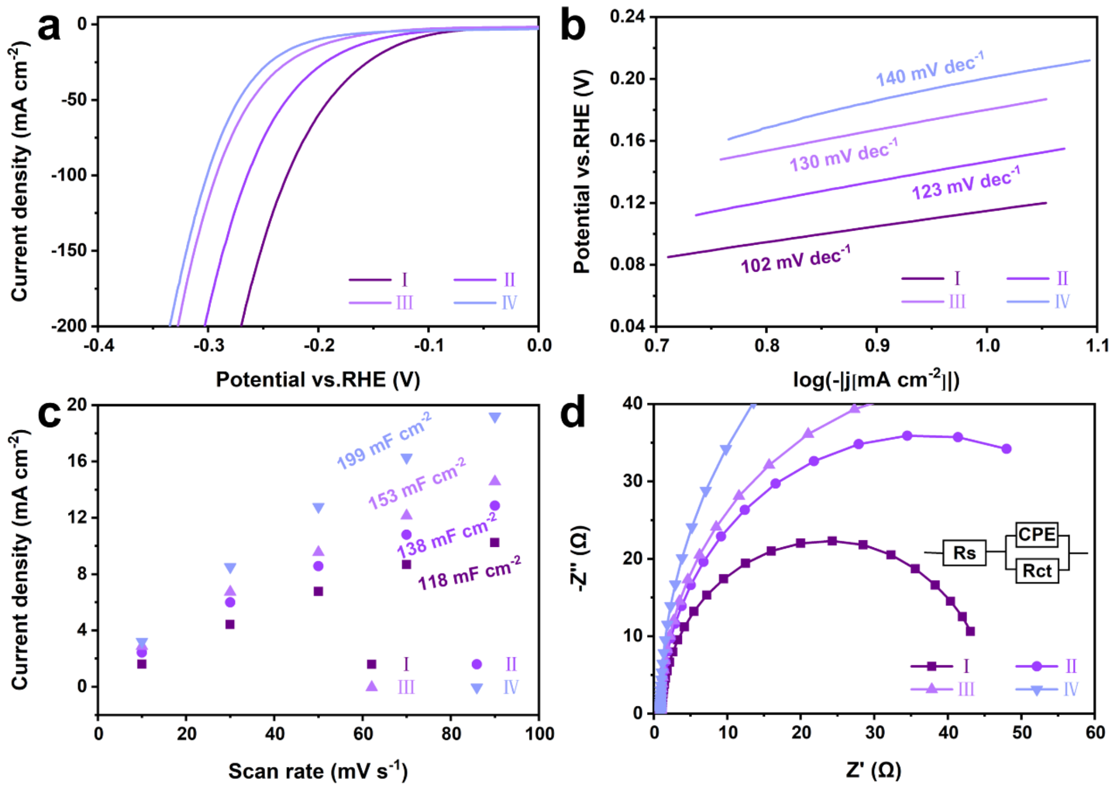

Figure S17 (a) Polarization curves, (b) Tafel plots, (c) C_{dl} at different scan rates, and (d) Nyquist plots of Ru-MoS_{2-x}-CoS_2/CC-80 (I), Ru-MoS_{2-x}-CoS_2/CC-240 (II), Ru-MoS_{2-x}-CoS_2/CC-280 (III), and Ru-MoS_{2-x}-CoS_2/CC-320 (IV). The electrolyte is N_2-saturated 1 M KOH.

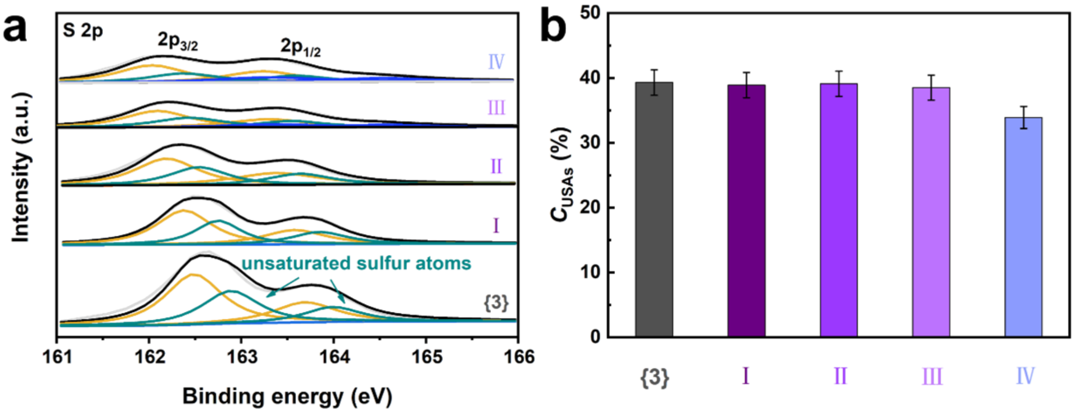

Figure S18 (a) S2p high-resolution XPS spectra and (b) concentration of unsaturated sulfur atoms (C_{USAs}) of Ru-MoS_2/CC ({3}), Ru-MoS_{2-x}-CoS_2/CC-80 (I), Ru-MoS_{2-x}-CoS_2/CC-240 (II), Ru-MoS_{2-x}-CoS_2/CC-280 (III), and Ru-MoS_{2-x}-CoS_2/CC-320 (IV).

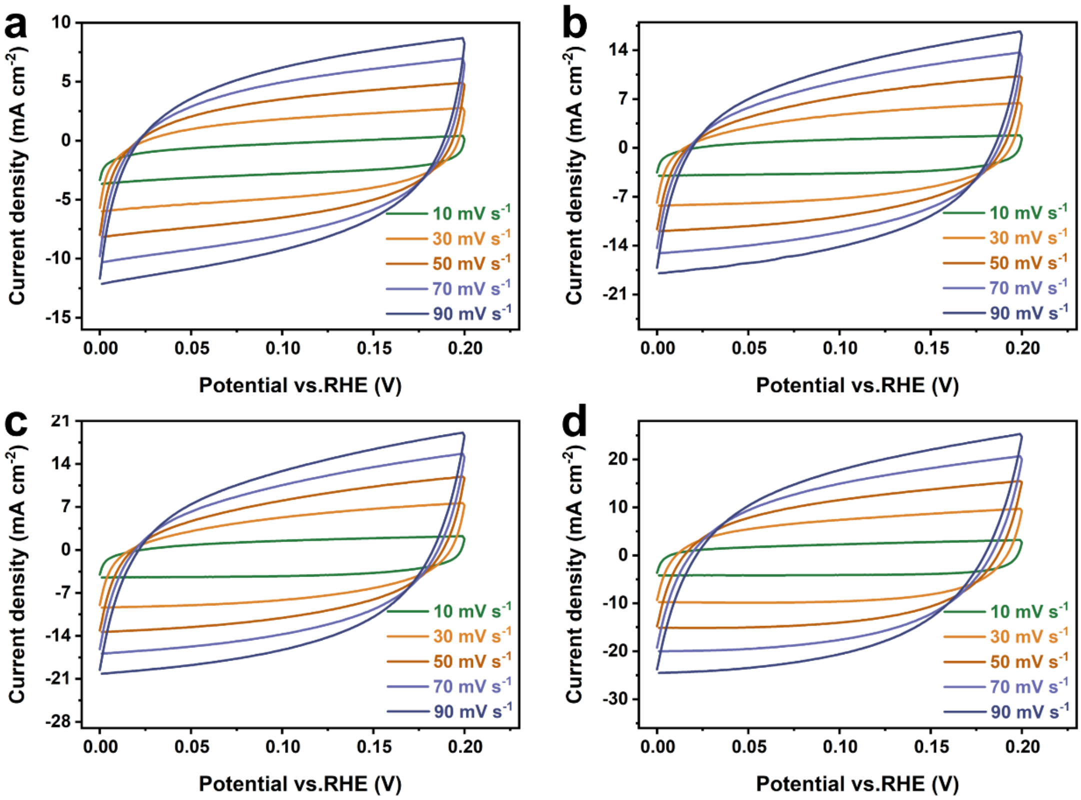

Figure S19 CV curves of (a) Ru-MoS_{2-x}-CoS_2/CC-80, (b) Ru-MoS_{2-x}-CoS_2/CC-240, (c) Ru-MoS_{2-x}-CoS_2/CC-280 and (d) Ru-MoS_{2-x}-CoS_2/CC-320. The scan rates are 10, 30, 50, 70 and 90 mV/s, respectively. The electrolyte is N_2-saturated 1 M KOH.

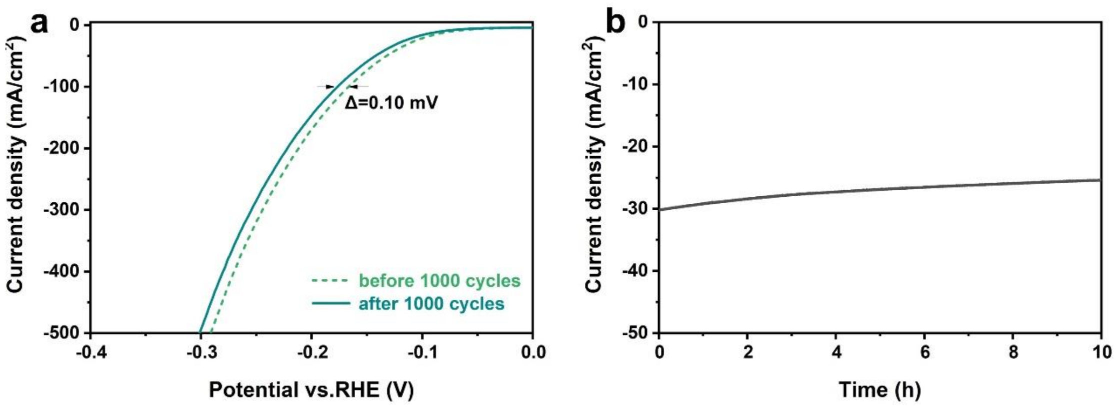

Figure 20 (a) Polarization curves of Ru-MoS_{2-x}-CoS_2/CC before and after 1000 cycles for a durability test, (b) time-dependent current density curve for Ru-MoS_{2-x}-CoS_2/CC under a static overpotential for 10 h.

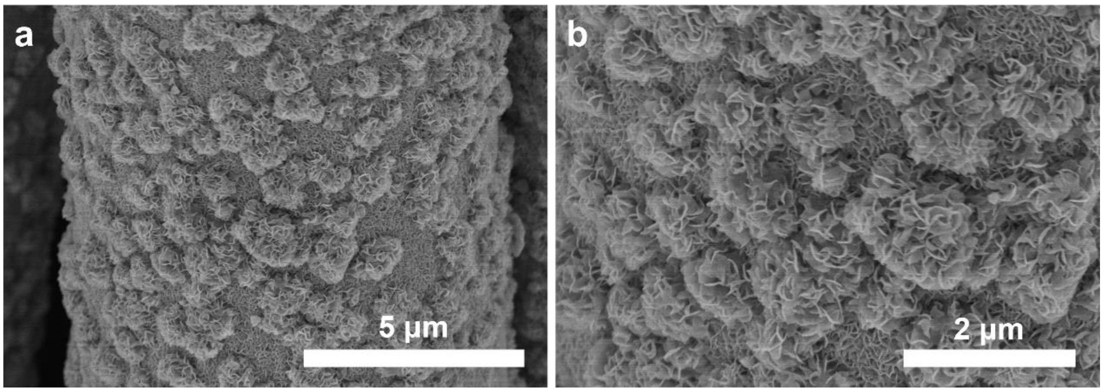

Figure 21 SEM images of Ru-MoS_{2-x}-CoS_2/CC after 1000 cycles.

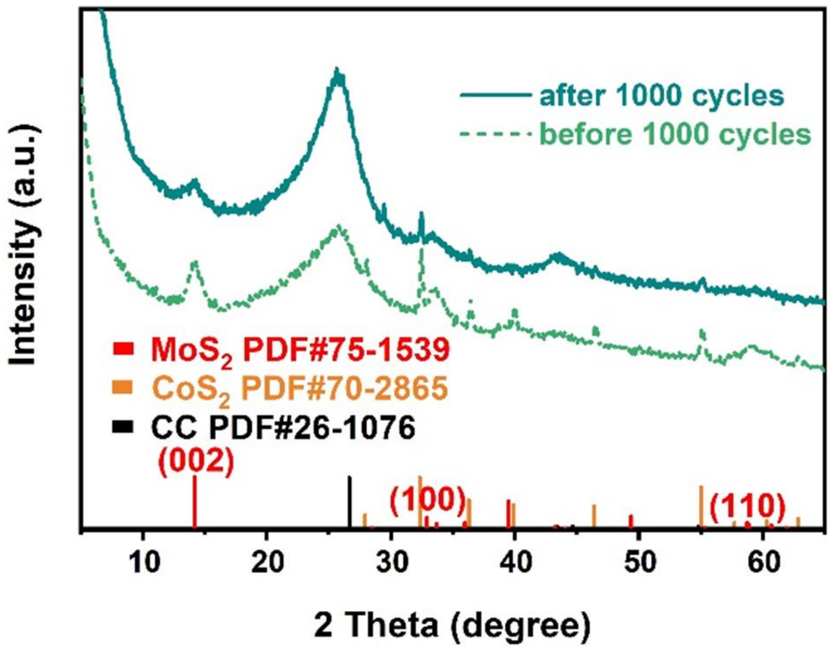

Figure 22 XRD pattern of Ru-MoS_{2-x}-CoS_2/CC before and after 1000 cycles.

Table S1 Molar ratio of Mo^{3+} to Mo^{4+}, atom ratio of S to Mo, and $C_{S\text{-vacancy}}$ of the related samples.

Samples	Mo^{3+} : Mo^{4+}	S : Mo	$C_{S\text{-vacancy}}$ (%)
MoS_2/CC [a)]	0.92	2 [b)]	0
MoS_2-CoS_2/CC	1.02	1.88	6.0
Ru-MoS_{2-x}-CoS_2/CC-1.0	1.11	1.84	7.9
Ru-MoS_{2-x}-CoS_2/CC	1.35	1.66	17.1
Ru-MoS_{2-x}-CoS_2/CC-7.0	1.30	1.72	14.0
Ru-MoS_{2-x}-CoS_2/CC-10.0	1.22	1.75	12.5
Ru-MoS_{2-x}-CoS_2/CC-30.0	1.19	1.76	12.0

[a)] At the volume of $RuCl_3$ solution (5 mmol L^{-1}) = 0.00 mL, the as-synthesized samples are MoS_2/CC. [b)] Atom ratio of S to Mo of MoS_2/CC is 1.89 : 1.

In addition, the atom ratio of S to Mo for MoS_2-CoS_2/CC is the atom ratio of S minus double Co to Mo which is abbreviated as [(S – 2Co)/Mo]. The atom ratio of S to Mo for all Ru-MoS_{2-x}-CoS_2/CC samples is the atom ratio of S minus double (Co + Ru) to Mo which is abbreviated as [(S – 2Co – 2Ru)/Mo].

Table S2 Atom ratio of S to Mo, and $C_{S\text{-vacancy}}$ of the related samples.

Samples	S : Mo	$C_{S\text{-vacancy}}$ (%)
Ru-MoS_2/CC-1.0	1.96	2.1
Ru-MoS_2/CC	1.94	3.1
Ru-MoS_2/CC-7.0	1.91	4.7
Ru-MoS_2/CC-10.0	1.86	6.8
Ru-MoS_2/CC-30.0	1.79	10.5

The atom ratio of S to Mo for all Ru-MoS_2/CC samples is the atom ratio of S minus double Ru to Mo which is abbreviated as [(S – 2Ru)/Mo].

Table S3 Atom ratio of S to Mo, and $C_{S\text{-vacancy}}$ of the related samples.

Samples	S : Mo	$C_{S\text{-vacancy}}$ (%)
Ru-MoS_{2-x}-CoS_2/CC-80	1.69	15.5
Ru-MoS_{2-x}-CoS_2/CC-240	1.63	18.5
Ru-MoS_{2-x}-CoS_2/CC-280	1.51	24
Ru-MoS_{2-x}-CoS_2/CC-320	1.45	27.5
MoS_2-CoS_2/CC-80	1.91	4.5
MoS_2-CoS_2/CC-240	1.87	6.5
MoS_2-CoS_2/CC-280	1.84	8.1
MoS_2-CoS_2/CC-320	1.79	11.5

Fabrications of MoS_2-CoS_2/CC-80, MoS_2-CoS_2/CC-240, MoS_2-CoS_2/CC-280, and MoS_2-CoS_2/CC-320 are almost the same as those of Ru-MoS_{2-x}-CoS_2/CC-80, Ru-MoS_{2-x}-CoS_2/CC-240, Ru-MoS_{2-x}-CoS_2/CC-280, and Ru-MoS_{2-x}-CoS_2/CC-320 except for the absence of $RuCl_3$ solution.

In addition, the atom ratio of S to Mo for all Ru-MoS_{2-x}-CoS_2/CC samples is [(S – 2Co – 2Ru)/Mo]; the atom ratio of S to Mo for all MoS_2-CoS_2/CC samples is [(S – 2Co)/Mo].
